# Microfluidic technologies for wearable and implantable biomedical devices

**DOI:** 10.1039/d5lc00499c

**Published:** 2025-08-13

**Authors:** Ziheng Wang, Ankit Shah, Hyowon Lee, Chi Hwan Lee

**Affiliations:** a School of Mechanical Engineering, Purdue University West Lafayette IN 47907 USA lee2270@purdue.edu; b Weldon School of Biomedical Engineering, Purdue University West Lafayette IN 47907 USA hwlee@purdue.edu; c Center for Implantable Devices, Purdue University West Lafayette IN 47907 USA; d School of Materials Engineering, Purdue University West Lafayette IN 47907 USA; e Elmore Family School of Electrical and Computer Engineering, Purdue University West Lafayette IN 47907 USA

## Abstract

Microfluidic technologies are transforming wearable and implantable biomedical devices by enabling precise, real-time analysis and control of biofluids at the microscale. Integrating soft, biocompatible materials with advanced sensing and fabrication techniques, these systems offer promising solutions for continuous health monitoring, targeted drug delivery, and responsive therapeutics. This review outlines critical design considerations, material strategies, and fluid handling mechanisms essential for device performance and biocompatibility. We systematically examine key fabrication approaches—including soft lithography, 3D printing, laser micromachining, and textile-based methods—highlighting their advantages and limitations for wearable and implantable applications. Representative use cases such as sweat analysis, interstitial fluid sampling, ocular diagnostics, wound monitoring, and *in vivo* therapeutic systems are explored, alongside current challenges in long-term stability, power management, and clinical translation. Finally, we discuss future directions involving bioresorbable materials, AI-assisted diagnostics, and wireless integration that may drive the next generation of personalized microfluidic healthcare systems.

## Abstract 2

This review highlights how microfluidic technologies advance wearable and implantable biomedical devices for real-time health monitoring and targeted therapy, while addressing materials, fabrication, applications, and clinical translation challenges.

## Graphical abstract

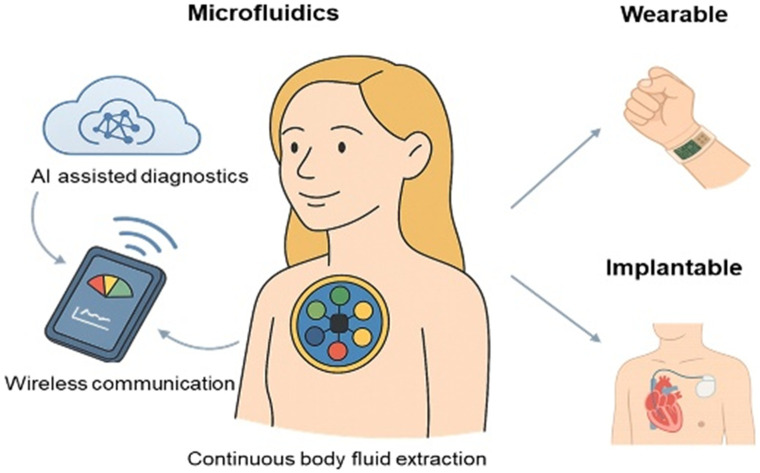

## 1. Introduction

**Fig. 1:**
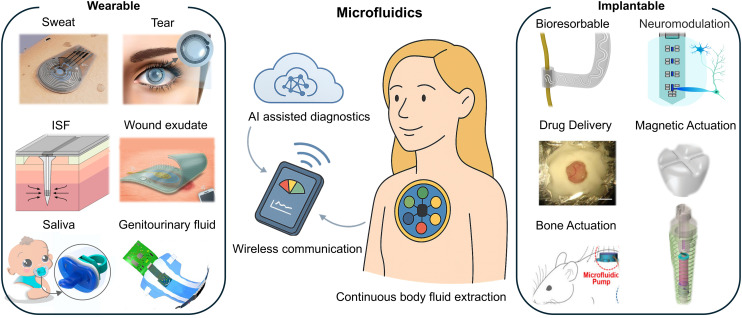
Overview of wearable and implantable microfluidic systems for continuous health monitoring. Reproduced in part with permission from ref. 20, copyright 2024 Wiley-VCH GmbH; ref. 218, copyright 2023 Elsevier; ref. 204, copyright 2025 Elsevier; ref. 231, copyright 2021 American Association for the Advancement of Science; ref. 209, copyright 2019 American Chemical Society; ref. 26, copyright 2022 American Chemical Society; ref. 170, copyright 2022 American Association for the Advancement of Science; ref. 172, copyright 2023 Frontiers; ref. 166, copyright 2014 Springer Nature; ref. 173, copyright 2024 Wiley-VCH GmbH; ref. 162, copyright 2020 MDPI.

Microfluidic devices are miniaturized systems engineered to precisely manipulate microliter- to nanoliter-scale fluid volumes through microscale channels.^1–3^ These platforms offer substantial benefits, including reduced reagent and sample consumption, faster assay times, enhanced analytical sensitivity, and portable operation.^3,4^ These advantages have led to widespread implementation in point-of-care diagnostics, therapeutic monitoring, and lab-on-chip platforms.^5–7^ The continued miniaturization of healthcare systems has driven the evolution of microfluidic technologies toward wearable and implantable formats capable of real-time analysis, seamless data transmission, and closed-loop feedback, all while maintaining user comfort and system reliability.^8–10^ As illustrated in Fig. 1, these developments have enabled a range of wearable and implantable microfluidic systems, which interface seamlessly with wireless communication modules and AI-assisted diagnostics to deliver dynamic health insights.

Recent progress in material engineering, microfabrication, and biosensor integration has significantly expanded the utility of microfluidic devices in biomedical applications.^11^ Soft, biocompatible materials such as elastomers, hydrogels, and stretchable polymers allow close mechanical conformity to the skin or internal tissues, minimizing irritation and enabling long-term use.^12,13^ These substrates provide the necessary flexibility and permeability for wearable systems while supporting chemical functionalization and fluidic control.^14^ Concurrently, advanced fabrication techniques—including soft lithography, 3D printing, laser micromachining, and micro-milling—allow for precise control over channel geometries, fluid dynamics, and reagent localization, thereby enhancing analytical resolution and enabling multifunctional integration.^15,16^ The addition of highly selective biosensors within microfluidic architectures further enables multiplexed biomarker detection and targeted therapy, offering clinically actionable insights for early disease detection, chronic disease management, and closed-loop treatment systems.^10,17^

Wearable microfluidic devices, designed for external applications, provide non-invasive or minimally invasive means of physiological monitoring and therapeutic delivery.^17^ These systems enable continuous and personalized physiological assessments through integration with biofluids such as sweat, saliva, tears, interstitial fluid (ISF), wound exudate and genitourinary fluid.^18–20^ For instance, epidermal microfluidic patches have been developed for sweat-based monitoring of dehydration, electrolyte imbalances, and glucose levels in diabetic patients.^21^ Lactate sensors support athletic performance tracking, while salivary diagnostics have been explored for monitoring stress hormones and oral health markers.^22^ In respiratory applications, smart face masks integrated with microfluidics analyze exhaled droplets to detect airborne pathogens.^23^ Tear-based biosensors embedded within contact lenses facilitate intraocular pressure and glucose monitoring, relevant for glaucoma and diabetes management.^24^ Wound-monitoring devices offer dynamic tracking of pH, temperature, and infection biomarkers, improving clinical outcomes in chronic wound care.^25^ Additionally, smart textiles and diapers embedded with microfluidic systems have been developed for infant care and incontinence monitoring.^26^ These wearable formats not only enable timely interventions but also provide data continuity for longitudinal health tracking.^17^

Implantable microfluidic devices function within the body, offering real-time monitoring and targeted therapy with minimal patient burden.^10,27^ These platforms enable continuous sampling of internal fluids such as blood, cerebrospinal fluid, or interstitial fluid, facilitating closed-loop control of chronic conditions.^28^ For example, implantable insulin delivery systems with microfluidic regulation offer precise glucose-responsive dosing, reducing the burden of diabetes management.^29^ Microfluidic neural probes facilitate the localized delivery of neuroactive compounds and simultaneous monitoring of neurotransmitter fluctuations, offering tools for treating neurological disorders such as Parkinson's disease.^30^ Advances in miniaturization, minimally invasive implantation techniques, and long-term biocompatible materials have substantially improved the safety, reliability, and longevity of such devices, bringing them closer to routine clinical translation.^31^

Despite significant progress, the widespread adoption of wearable and implantable microfluidic technologies is constrained by several challenges.^17^ Long-term sensor stability, biofouling resistance, mechanical durability, and continuous power supply remain major concerns.^31^ For wearable systems, adhesion to dynamic skin surfaces and susceptibility to environmental interferences such as sweat pH or temperature variations can affect signal fidelity.^32^ Implantable devices must contend with immune responses, fibrosis, and biofluid ingress, all of which can deteriorate sensing accuracy or therapeutic function over time.^28^ Miniaturization of multi-modal systems while maintaining performance, as well as the integration of secure wireless communication and cloud-based analytics, is essential for broader clinical deployment.^32^ Moreover, regulatory compliance, ethical data handling, and large-scale manufacturing remain as barriers that must be addressed through interdisciplinary collaboration.

**Fig. 2:**
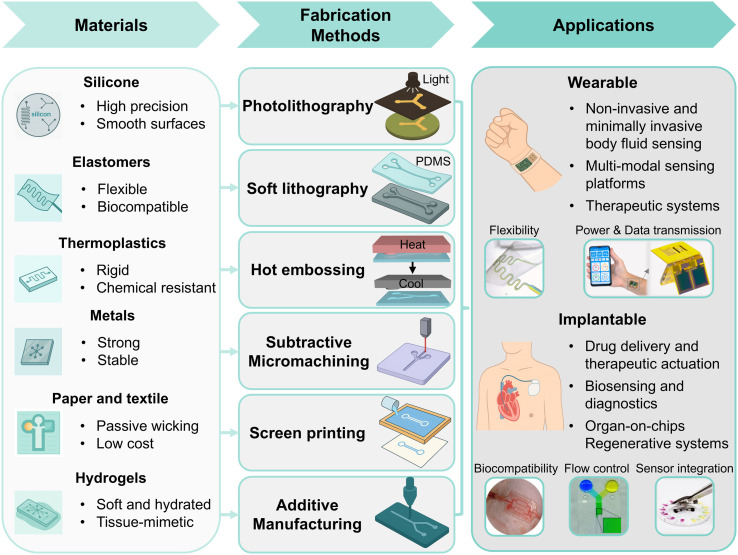
Material–method–application decision framework for wearable and implantable microfluidic devices. Reproduced in part with permission from ref. 34, copyright 2017 American Chemical Society; ref. 39, copyright 2023 Springer Nature; ref. 36, copyright 2016 Springer Nature; ref. 35, copyright 2021 Royal Society of Chemistry; ref. 188, copyright 2019 American Association for the Advancement of Science.

This review presents a comprehensive overview of recent advancements in wearable and implantable microfluidic systems, highlighting how design considerations guide material selection and inform compatible fabrication methods for targeted biomedical applications (Fig. 2). Key topics include fluid manipulation mechanisms, sensing modalities, and material selection parameters that collectively enhance device functionality, biocompatibility, and user comfort. A comparative analysis of fabrication methods—such as soft lithography, 3D printing, and screen printing—is provided, with attention to their resolution, throughput, and scalability. Application areas span wearable platforms for sweat analysis, wound monitoring, and ocular diagnostics, as well as implantable systems for precision drug delivery and chronic disease management. Critical challenges—including material degradation, long-term biostability, power supply limitations, and regulatory compliance—are discussed in the context of real-world deployment. Future directions highlight the integration of self-sustaining power sources, advanced biomaterials, and AI-assisted data analytics to enable intelligent, autonomous health monitoring. By addressing both the transformative potential and practical barriers, this review aims to inform and inspire continued innovation in microfluidic technologies that support personalized, preventive, and responsive healthcare.

## 2. Design considerations for microfluidic devices

The development of microfluidic devices for wearable and implantable applications necessitates the careful integration of material science, fluid mechanics, sensor engineering, and system-level design.^31,33^ Key considerations include material flexibility and biocompatibility, efficient biofluid handling, sensor and electronic integration, power sustainability, and reliable data communication.^34,35^ Material selection not only dictates mechanical resilience and skin or tissue compatibility but also influences resistance to biofouling and degradation.^9,36^ Fluid handling mechanisms—whether passive or active—must ensure precise transport and sampling under physiological conditions. Sensor integration is essential for real-time, *in situ* detection with minimal interference.^37^ Compact power solutions and wireless communication modules further enable autonomous operation and connectivity with external healthcare platforms.^38,39^ The following sections detail the specific design requirements and challenges for wearable and implantable microfluidic systems.

### 2.1. Material considerations

Wearable on-skin and implantable *in vivo* microfluidic devices share fundamental material requirements: both demand biocompatibility, chemical inertness, and mechanical suitability for microscale fluid manipulation. However, distinct application contexts impose divergent constraints. Wearable devices must withstand environmental exposure—such as air, moisture, and mechanical deformation—while ensuring skin comfort and long-term wearability.^40^ In contrast, implantable systems function within enclosed physiological environments, necessitating materials with prolonged stability, resistance to biofouling, and minimal degradation over time.^31^

Recent advances in materials science have addressed these challenges across both domains.^41–43^ Developments in stretchable nanocomposites, conductive and stimuli-responsive hydrogels, and biodegradable polymers have enabled improved integration with biological tissues and expanded the functional versatility of microfluidic platforms.^44–46^Table 1 summarizes representative materials employed in wearable and implantable systems, which are further discussed in the following sections.

**Table 1:** Summary of representative materials used in wearable and implantable microfluidic devices

Material category	Representative materials	Advantages	Limitations	Application	Ref.
Elastomers	PDMS, Ecoflex	Highly flexible; stretchable; conformal contact; biocompatible; transparent; microfabrication-compatible	Limited solvent resistance; gas permeability	Wearable & implantable	43, 54
Hydrogels	PEG, alginate, PAA, PVA, HA	Soft, hydrated, tissue-like properties; stimuli-responsive; drug delivery capability	Drying; swelling and mechanical instability	Wearable & implantable	56, 58, 61
Thin-film polymers	Parylene C, polyimide, PET	High chemical stability; MEMS compatible; excellent encapsulation	Not stretchable, requires hybridization with soft layers	Wearable & implantable	68, 70, 71
Thermoplastics	PMMA, COC, PC, PEEK, PTFE	Strong, mechanical stable; good optical clarity; chemical resistant; precise machining	Rigid; limited use in conformal or dynamic applications	Primarily implantable	55, 64
Fibrous materials	Cellulose paper, cotton, textile thread	Light weight; breathable; passive wicking; low-cost; easy fabrication	Fragile; not sterilizable; not suitable for implantation	Primarily wearable	63, 128, 158
Inorganic materials	Silicon, glass, titanium, steel	High strength; high precision; MEMS-compatible; long term durability	Rigid, mechanical mismatch with soft tissues; requires encapsulation	Selective use	76, 105
Bioresorbable materials	PLGA, PLA, magnesium alloys, bioactive glass	Biodegradable; avoids secondary surgeries; suitable for transient implants	Degradation-dependent stability; complex degradation tuning	Implantable	31, 79

#### 2.1.1. Material considerations for wearable microfluidics

Wearable microfluidic devices demand materials that are not only biocompatible but also capable of maintaining mechanical integrity and functional reliability under continuous motion and environmental exposure.^47^ A primary design objective is to achieve intimate, conformal contact with the skin by replicating its mechanical characteristics.^40,48^ Human skin exhibits a Young's modulus typically ranging from ∼0.05 to 2 MPa, depending on both anatomical region and depth within the skin layers. Its stretchability also varies with location, with jointed regions such as the elbow and knee capable of sustaining strains up to 60–70%, while flatter areas like the forearm or abdomen typically experience lower strains in the range of 20–30% during normal movement.^49–51^ To accommodate such deformation, wearable microfluidics employ materials that balance softness, stretchability, and durability, ensuring stable operation during prolonged daily use.

Thermoplastics such as poly(methyl methacrylate) (PMMA) and cyclic olefin copolymer (COC) offer advantages in optical transparency, mechanical strength, and compatibility with scalable manufacturing processes. However, their inherent rigidity and limited conformability restrict their suitability for skin-mounted, stretchable systems.^52,53^ In contrast, elastomeric materials such as polydimethylsiloxane (PDMS) and Ecoflex provide a deformable, conformal interface capable of accommodating skin motion while supporting the integration of fluidic networks and electronic components. Their optical clarity and permeability are beneficial for colorimetric sensing and sweat evaporation, though limitations in solvent resistance and large-scale manufacturability remain.^33,54^

To overcome these trade-offs, recent efforts have focused on hybrid materials, particularly soft thermoplastic elastomers (TPEs) such as styrene-ethylene-butylene-styrene (SEBS) copolymers, thermoplastic polyurethanes (TPUs), and thermoplastic styrenic block copolymers (TPSs). These materials combine the mechanical flexibility of elastomers with the processability of thermoplastics, enabling stretchable microfluidic architectures that can be mass-produced while maintaining skin compatibility and structural resilience.^55^

Hydrogels and adhesive polymers serve complementary roles in wearable systems by improving adhesion, comfort, and functional responsiveness.^17^ Hydrogel-based adhesives—such as those composed of polyacrylate or polyurethane—offer soft, hydrated interfaces that conform to the microtopography of skin while minimizing irritation.^56^ Their high water content not only enhances comfort but can also support sensing functionality by responding to analyte-induced swelling, ionic conductivity, or colorimetric change.^57,58^ However, conventional hydrogels are prone to dehydration and mechanical fatigue during prolonged use. To enhance their stability and longevity, strategies such as increasing crosslinking density, integrating thermosensitive polymers, and tuning polymer–water interactions have been employed.^59–61^

For low-cost, breathable, and disposable wearable platforms, fiber-based microfluidic systems—such as paper- and textile-based substrates—are gaining popularity. These materials utilize intrinsic porosity and capillary wicking properties to enable passive biofluid transport without external pumps.^9^ Microfluidic paper-based analytical devices (μPADs) and thread-based channels can perform colorimetric or electrochemical sensing in a simple and scalable format.^62,63^ Although limited in structural precision compared to lithographically patterned devices, these systems prioritize user comfort, manufacturability, and cost-effectiveness.^8^

Finally, multilayer composite structures are frequently employed to enhance device performance. Thin polymer films such as polyethylene terephthalate (PET) provide flexible, dimensionally stable substrates for microfluidic patterning *via* printing or laser ablation.^64^ Coupled with biocompatible adhesives, these layers form epidermal tape-like devices that adhere securely during active use, ensuring stable microfluidic operation under mechanical stress.^65^

#### 2.1.2. Material considerations for implantable microfluidics

Implantable microfluidic devices face an even stricter set of material considerations because they reside inside the body for extended periods. The priority is biocompatibility and biosafety: materials must not provoke chronic inflammation or toxicity, and they should resist corrosion or degradation in the warm, aqueous, enzymatic conditions of the body. Polymers such as PDMS, polyethylene glycol (PEG), and polyether ether ketone (PEEK), along with other inert materials, are frequently used to construct or encapsulate implantable microfluidic devices due to their biocompatibility and stability. These materials can conform to soft tissues—such as the brain, heart, or internal organs—with minimal irritation. However, they may be prone to permeability issues and long-term degradation, necessitating additional surface modifications or protective coatings.^66,67^

Thin-film polymer substrates play a dual role in both encapsulation and structural design of implantable microfluidic devices.^68^ Parylene-C, a chemically vapor-deposited polymer, is a leading material due to its exceptional biocompatibility, pinhole-free hermetic sealing, and compatibility with microfabrication.^69–71^ It can serve as a conformal coating to improve the barrier properties of elastomers or function as a standalone structural layer for microchannels and membranes.^72^ In a recent work, flexible parylene membranes have been used to seal intraocular microfluidic pressure sensors, ensuring leak-free operation under ocular pressure fluctuations.^73^ Similarly, polyimide is commonly employed in neural implants, where its flexibility and mechanical robustness support both microfluidic conduits and embedded electrodes for chronic implantation.^74^

Rigid materials such as metals and microfabricated silicon play a critical role in implantable devices requiring structural precision, mechanical durability, or integration with electronic components.^75,76^ Metals like titanium and stainless steel provide strong mechanical strength and long-term biostability, and are routinely used in reservoirs, support frames, or sealing elements. At the microscale, silicon provides exceptional fabrication precision and compatibility with MEMS processes, enabling the construction of intricate fluidic channels, valves, and sensing elements. However, due to their intrinsic stiffness, these materials are often encapsulated in soft, biocompatible coatings to mitigate mechanical mismatch and reduce tissue irritation.^77^ A notable example is early-generation MEMS drug delivery implants, which integrated silicon micro-reservoirs within titanium housings and medical-grade silicone encapsulants to ensure both mechanical robustness and biocompatibility.^78^

Emerging trends in implantable microfluidics include the integration of dynamic, resorbable, or biointeractive materials.^79^ Hydrogels are increasingly incorporated as compliant, tissue-mimicking interfaces or bioactive scaffolds. Meanwhile, biodegradable polymers such as poly(lactic-*co*-glycolic acid) (PLGA), poly(anhydrides), and other bioresorbable materials enable the fabrication of transient implants.^31,80^ These devices are engineered to function over a defined therapeutic window—such as for drug delivery or tissue regeneration—and subsequently degrade harmlessly *in situ*, eliminating the need for surgical retrieval. A representative example is a PLGA-based microfluidic drug delivery implant that achieved controlled release over several weeks before undergoing complete resorption *in vivo*, demonstrating the feasibility of biodegradable platforms for temporary therapeutic applications.^81^ Balancing degradation kinetics with mechanical integrity remains a major design challenge, particularly for systems requiring precise fluid handling or long-term structural stability.

### 2.2. Engineering considerations

Designing microfluidic devices for wearable and implantable use involves shared engineering demands—miniaturization, precise fluid handling, biocompatibility, and reliable sealing—alongside distinct contextual challenges. Both systems must autonomously manage fluids at microliter to nanoliter scales and integrate compact, low-power electronics for sensing and communication.^82^ Wearables emphasize simplicity, passive flow, and user robustness, while implants require long-term stability, active control, and surgical compatibility.^83^ Engineers must also address thermal regulation and mechanical safety. The following sections outline key strategies for fluid management, power integration, and device reliability, highlighting recent advances tailored to wearable and implantable microfluidic platforms.

#### 2.2.1. Engineering considerations for wearable microfluidics

Engineering wearable microfluidic devices require careful design to ensure reliable operation on the dynamic, irregular surface of the skin. These systems must effectively handle fluid sampling and transport while remaining flexible, unobtrusive, and compatible with motion.^17^ Fluid handling is often achieved using passive mechanisms, such as capillary-driven flow, hydrophilic and hydrophobic patterning or pressure from sweat glands, eliminating the need for external pumps.^84,85^ However, passive strategies can suffer from variability—differences in sweat rate or body location may influence sensor response, affecting reliability.^86^

To address this, researchers have introduced active fluid control elements in soft, conformal platforms. For example, Lin *et al.* developed a programmable epidermal system incorporating thermo-responsive hydrogel valves actuated by thin-film microheaters, enabling consistent and on-demand sweat routing.^87^ Active components are embedded using stretchable electronics, micro-actuators, or stimuli-responsive materials, which swell or shrink in response to external stimuli (*e.g.*, temperature or hydration). The method enabled dynamic control while maintaining mechanical compliance with the skin.

Sensor integration is another central challenge.^32^ Wearable microfluidics may use colorimetric detection (*e.g.*, pH- or glucose-sensitive dyes) or printed electrochemical sensors (*e.g.*, for lactate or sodium). These sensors must retain high sensitivity and selectivity while being stretchable and miniaturized. Achieving mechanical flexibility without compromising performance requires the use of stretchable conductive inks, soft interconnects, and miniaturized sensor chips. Encapsulation with breathable yet protective films enhances environmental robustness and safeguards reagents from contamination and mechanical damage.^88^

Power and communication modules must also conform to wearable constraints.^89^ Compact power sources such as thin-film batteries or energy-harvesting systems have been developed to reduce reliance on bulky components.^90,91^ Notably, triboelectric nanogenerators (TENGs) and piezoelectric elements can convert biomechanical motion (*e.g.*, walking or subtle skin deformation) into usable power, sufficient for low-duty-cycle sensors.^92,93^ Some devices combine energy harvesting and sensing—for example, a TENG that acts as both motion-powered generator and pressure sensor. Wireless data transmission is typically achieved *via* bluetooth low energy (BLE) for continuous monitoring or near field communication (NFC) for intermittent readouts.^94^

Overall, wearable microfluidic systems integrate soft materials, autonomous fluid handling, embedded sensors, and wireless modules to achieve high-performance biosensing in a user-friendly, skin-conformal form.

#### 2.2.2. Engineering considerations for implantable microfluidics

Implantable microfluidic devices must deliver reliable, long-term performance within complex *in vivo* environments while ensuring patient safety. In contrast to wearable systems, passive fluid handling is typically insufficient, necessitating the use of active fluid control mechanisms to precisely manage flow, sampling, and drug delivery.^28^ Micro-electromechanical systems (MEMS)-based micropumps—such as piezoelectric diaphragm pumps and electroosmotic actuators—are commonly integrated with microvalves to achieve controlled dosing.^95^ For example, implantable insulin pumps modulate drug release in response to sensed glucose levels, creating a closed-loop feedback system.^96^ Safety-critical features, such as pressure relief valves, are incorporated to prevent overdosing or device failure.^97^

To enable continuous monitoring, biosensors are embedded within the implantable platforms to measure physiological markers such as glucose, pH, and electrolytes. However, sensor reliability is challenged by biofouling, resulting from protein adsorption or cellular overgrowth.^31^ This is mitigated by antifouling strategies, including PEGylated surfaces, zwitterionic coatings, or semi-permeable membranes that isolate sensing elements.^98^ Advances in MEMS fabrication have further enabled the integration of miniaturized, high-resolution sensors into microfluidic systems, enhancing diagnostic accuracy and enabling localized biochemical analysis in real time.

Power management remains a central engineering hurdle, as surgical intervention is often required to replace depleted batteries. While some short-term or low-power implants still utilize compact, sealed batteries, long-term devices increasingly rely on wireless power transfer.^31^ Techniques such as inductive coupling—where an internal coil receives power from an external transmitter—are widely used in cochlear implants, neural stimulators, and infusion pumps.^99^ In deeper tissues, ultrasound-based power transfer offers better penetration, while energy harvesting technologies, including piezoelectric or triboelectric nanogenerators, aim to capture biomechanical or biochemical energy to sustain ultra-low-power operation.^100–102^

Reliable wireless communication is equally critical for enabling real-time monitoring and remote diagnostics. Technologies such as bluetooth low energy (BLE), near-field communication (NFC), and custom RF telemetry allow implants to transmit data through tissue to external receivers.^103^ Given the sensitivity of health data, secure data handling is imperative. End-to-end encryption, robust authentication protocols, and emerging blockchain-integrated frameworks are being developed to safeguard medical information and ensure data integrity within connected healthcare ecosystems.

## 3. Fabrication techniques for microfluidic devices

The fabrication of wearable and implantable microfluidic devices requires precise methods to ensure structural integrity, functional reliability, and biocompatibility. These techniques must accommodate diverse material properties while enabling the seamless integration of microchannels, biosensors, and electronic components.^16^ The choice of fabrication method depends on the intended application, resolution requirements, and material compatibility. Various fabrication techniques have been developed to meet these demands.^104^ The following sections give detailed introductions to key fabrication methods and present specific examples of these fabrication techniques for wearable and implantable microfluidic devices, highlighting their advantages, limitations, and potential improvements for future biomedical applications.

### 3.1. Key fabrication methods for wearable and implantable microfluidics

Wearable and implantable microfluidic devices require fabrication techniques that accommodate miniaturization, flexibility, and biocompatibility. Early lab-on-chip devices were typically made using photolithography and etching on silicon or glass, yielding precise microchannels but with high cost and rigidity.^105^ To overcome these limitations, a range of newer methods—such as polymer casting (soft lithography), laser micromachining, hot embossing, 3D printing, and fiber-based microfluidics—have been developed.^106^ Each method offers distinct advantages and challenges, and careful integration and encapsulation are needed to translate microfluidic systems into wearable or implantable form factors.

#### 3.1.1. Photolithography for high-precision microfluidics

Photolithography is a planar microfabrication technique adapted from the semiconductor industry to define microscale fluidic patterns on substrates. In this process, a photosensitive resist (*e.g.* SU-8) is coated on a rigid substrate (silicon, glass, *etc.*), exposed through a mask, and developed to create raised channel structures.^107^ Early microfluidic “lab-on-a-chip” devices in the 1990s were fabricated by photolithography and chemical etching on silicon or glass. Photolithography enables the fabrication of high-resolution features down to the sub-micron scale, with smooth sidewalls and excellent surface quality, making it ideal for applications requiring precise micro- and nano-patterning.^16^ However, traditional photolithographic fabrication requires cleanroom facilities and rigid materials, making it expensive, time-consuming, and poorly suited for rapid prototyping or flexible wearable designs. These limitations have motivated the use of softer materials and alternative fabrication methods in recent years.^108^

#### 3.1.2. Soft lithography for polymer-based microfluidics

Soft lithography refers to a collection of molding techniques that replicate microscale patterns using elastomeric materials.^109^ The most common form is PDMS casting, which has been widely used for microfluidic prototyping due to its low cost and simplicity. Typically, a liquid PDMS mixture (base prepolymer and curing agent) is poured onto a master mold—typically created *via* photolithography (*e.g.*, SU-8 patterned on silicon)—and thermally cured. The elastomeric stamp is then peeled off, producing a PDMS layer with defined microchannels.^110^ This PDMS layer can be bonded to glass or another PDMS piece (often by oxygen plasma treatment) to form enclosed microfluidic channels.^111^ Soft lithography allows rapid prototyping of microfluidic designs with feature sizes down to a few microns, replicating the mold's geometry with high fidelity and inheriting its surface smoothness and resulting in a transparent and flexible device.^112^

Despite its widespread use, PDMS exhibits several limitations that hinder its application in long-term wearable or implantable systems. These include high permeability to gases and small molecules, propensity for solvent-induced swelling, mechanical deformation under stress, and susceptibility to aging-related degradation.^113^ To overcome these challenges, research is increasingly focused on alternative elastomers—such as polyurethane and thermoplastic elastomers—as well as surface treatments that improve chemical resistance and structural robustness, aligning soft lithography with the stringent demands of biomedical deployment.^67^

#### 3.1.3. Subtractive micromachining and hot embossing

Subtractive micromachining utilizes direct material removal to create microfluidic structures, encompassing techniques like laser and computer numerical control (CNC) micromachining. Laser micromachining utilizes focused laser beams (UV, picosecond, or femtosecond pulses) to ablate material directly, forming microchannels without molds or masks. This versatile and maskless technique supports rapid prototyping across diverse substrates (polymers, glass, metals) and enables complex channel designs.^114–116^ Typical commercial laser systems achieve feature resolutions around 10–20 μm, while advanced ultrafast lasers—such as femtosecond systems—can ablate features as small as ∼1–3 μm with careful control of pulse duration, beam focus, and scanning parameters.^105^ However, laser-formed channels often exhibit elevated surface roughness due to localized melting and redeposition of ablated material.^117^ Ongoing improvements in ultrafast laser technology and post-processing techniques, such as chemical polishing, are steadily enhancing microchannel surface quality.^118^

In contrast, CNC micromachining offers improved surface finish and tighter geometric control, particularly in polymer and metal substrates, making it a valuable complementary technique. It uses computer-controlled miniature endmills to mechanically cut microchannels and 3D features—such as pockets, chambers, and through-holes—directly into the substrate.^105^ Like laser ablation, it is a mold- and mask-free approach well-suited for rapid prototyping. However, heat generated during milling can lead to burr formation, and the small size of micro-tools makes tool wear difficult to monitor and failure harder to predict. Issues such as built-up edge formation, runout, and tool fragility can limit the minimum achievable feature size and compromise machining precision if not carefully controlled.^119^ Given these traits, subtractive techniques are most effective for rapid prototyping or for fabricating high-fidelity molds and master structures, which can then be employed in replication-based methods.

One such method is hot embossing, which replicates microchannel structures by pressing heated molds into thermoplastic substrates (*e.g.*, polycarbonate, PMMA). It efficiently reproduces precise microstructures suitable for scalable production, offering enhanced chemical resistance and mechanical robustness relative to PDMS.^120^ Nonetheless, precise control of temperature and pressure conditions is essential to mitigate residual stress and ensure uniformity. Innovations in mold fabrication and release coatings are progressively addressing these limitations, improving hot embossing's viability for mass-produced microfluidic devices.^121^

#### 3.1.4. Additive manufacturing

3D printing, or additive manufacturing, has emerged as a transformative fabrication method, enabling direct production of complex three-dimensional microfluidic architectures unattainable with planar lithographic methods.^122^ Various 3D printing modalities have been applied to microfluidics: for example, stereolithography (SLA/DLP) printers can cure resin with ∼10–100 μm resolution, inkjet or PolyJet printers can deposit intricate multi-material structures, and extrusion printers can lay down flexible filaments to form large microfluidic conduits.^123^ These methods enable intricate internal structures, vertical interconnects, and monolithic integrated features such as valves and mixers.^124,125^ This significantly shortens development cycles and lowers upfront costs. Modern 3D printers achieve resolutions down to tens of microns, with advanced two-photon polymerization pushing into sub-micron scales.^126^

Despite these advantages, challenges remain—particularly in achieving smooth internal channel surfaces. In general, 3D-printed microfluidic devices exhibit surface roughness in the range of ∼0.35 to 40 μm, depending on the printing modality and material, which often necessitates post-processing to ensure fluidic compatibility. While advanced techniques such as two-photon polymerization can achieve nanometer-scale surface quality (as low as 4–11 nm), their limited throughput restricts broader applicability. Nonetheless, 3D printing's design versatility, rapid iteration capability, and material adaptability (including biocompatible flexible resins) significantly benefit personalized wearable and implantable microfluidic device development.^127^

#### 3.1.5. Fabrication techniques for fibrous microfluidics

Fibrous microfluidics leverage porous, thread-like, or textile-based materials to transport fluids *via* capillary action, enabling the development of flexible, wearable, and low-cost fluidic platforms.^128^ Techniques such as wax printing, screen printing, inkjet printing, xurography, sewing, and embroidery provide scalable, accessible, and adaptable fabrication options.^129^ Wax printing involves selective deposition of hydrophobic wax onto cellulose substrates to define precise fluidic pathways, suitable for single-use applications. Similarly, screen printing uses stencil-guided deposition of robust hydrophobic inks onto textiles, offering scalability and compatibility with industrial manufacturing.^9^

Inkjet printing offers mask-free, precise patterning capabilities, ideal for complex geometries on flexible substrates. Xurography facilitates rapid prototyping through cutting and lamination of polymer films. This rapid prototyping approach is beneficial for straightforward designs requiring quick iterations.^129^ Sewing and embroidery techniques integrate hydrophilic or conductive threads directly into fabrics, creating embedded fluid channels and sensing networks with strong mechanical resilience and seamless textile integration.^62^

In fibrous microfluidics, fluid transport is governed by the porosity and fiber architecture of the material, rather than by defined channel walls. For example, liquid wicks along cellulose fibers in paper or through the core of braided threads. This results in higher microscale roughness and variability in flow paths compared to lithographically defined microchannels, but such irregularities are acceptable for capillary-driven transport and the relatively large sample volumes typical of these systems. Key advantages of fibrous platforms include mechanical flexibility—enabling integration with skin or textiles—and low fabrication cost. Printing techniques are easily adaptable to large-area or roll-to-roll manufacturing, making fibrous microfluidic devices well-suited for scalable, disposable, and wearable diagnostic applications.

#### 3.1.6. Comparison of fabrication methods

Fabrication strategies for wearable and implantable microfluidics must be aligned with functional priorities—namely precision, mechanical adaptability, and production scalability. While no single technique consistently excels across all these dimensions, understanding their respective trade-offs enables rational selection based on the specific demands of a given application.

For applications requiring high microscale fidelity—such as microneedle inlets or implantable microvalves—fabrication precision is critical to ensure predictable fluid dynamics and avoid clogging or dosing errors. Among available techniques, photolithography offers the highest resolution, enabling sub-micron feature definition. When combined with controlled etching processes such as reactive ion etching (RIE) or wet chemical etching, it can produce exceptionally smooth surfaces, with surface roughness (Ra) values typically below 10 nm on glass or silicon substrates.^16,130^ Soft lithography using PDMS replication provides slightly lower resolution, generally in the Sub-100-nm range, but achieves similarly smooth surfaces when high-quality master molds are used.^131^ Hot embossing enables high-resolution replication in thermoplastics, with surface roughness typically around 1 μm and reducible to tens of nanometers using precision-polished molds.^132,133^ Subtractive approaches such as CNC micromilling and laser ablation are suitable for rapid prototyping and mold fabrication, offering feature tolerances down to 1–3 μm and surface roughness values as low as 65 nm, though post-processing is often necessary to improve surface quality.^105^ While many 3D printing techniques exhibit limited precision for microscale applications, high-resolution methods such as stereolithography (SLA) can achieve feature sizes around 100–200 μm and channel surface roughness as low as ∼0.35 μm.^134^ Two-photon polymerization offers even finer resolution, enabling sub-micron features and producing optically smooth surfaces suitable for complex micro- and nano-fluidic structures.^135^

For devices interfacing with soft, deformable tissue—such as skin-mounted sensors or bioresorbable implants—fabrication methods must support materials that are both biocompatible and capable of withstanding region-specific strains without compromising function. Soft lithography enables microchannel patterning in elastomeric substrates like PDMS and TPU, which are well suited for relatively low-strain anatomical regions such as the forearm or chest. These materials conform to moderate skin deformation while preserving feature fidelity. For higher-strain regions such as joints, highly stretchable elastomers like Ecoflex are preferred, offering greater compliance under dynamic motion. Additive manufacturing provides further flexibility, enabling multilayered, anatomically customized structures using flexible resins or hydrogel-based bioinks that accommodate moderate deformation. Fiber- and textile-based approaches offer robust mechanical resilience and breathability, making them ideal for wearable applications over mobile or contoured surfaces where both stretchability and comfort are essential.^33,105^

When large-scale production and cost-efficiency are prioritized—such as in disposable diagnostics or therapeutic patches—fabrication methods must support high throughput and consistent yield. In this context, scalability generally implies the capability to transition from small-batch prototyping to continuous or high-volume production while maintaining reproducibility and quality. Techniques that are compatible with automation or roll-to-roll (R2R) processing are typically regarded as scalable, as they enable the fabrication of thousands to hundreds of thousands of units with minimal manual intervention. For example, hot embossing enables the reproduction of intricate microstructures in thermoplastics with short cycle times and high pattern fidelity, making it suitable for medium- to high-volume production. Injection molding is well-suited for mass production, allowing consistent replication of complex microfluidic components with tight dimensional control. Printing-based approaches, including screen printing and inkjet deposition, integrate readily with roll-to-roll systems, enabling continuous patterning on flexible substrates such as polymer films or paper—ideal for disposable or wearable formats. Wax printing, despite lower resolution, remains a popular choice for its simplicity, affordability, and compatibility with lateral flow and paper-based microfluidic devices. While less suited for high-volume manufacturing, 3D printing and micromilling remain indispensable for rapid prototyping, iterative design, and the fabrication of customized or small-batch devices.^136^

Table 2 summarizes the above-mentioned fabrication techniques for wearable and implantable microfluidic devices, comparing their respective advantages and limitations. As device complexity and functional integration increase, converging high-resolution fabrication with soft material compatibility and scalable production will be pivotal to advancing next-generation wearable and implantable microfluidic systems.

**Table 2:** Comparisons of the advantages and limitations for fabrication methods of wearable and implantable microfluidic devices

Fabrication methods	Materials	Advantages	Disadvantages	Ref.
Photolithography	• Silicon	• High precision	• Costly	9, 16, 107, 108
	• Glass	• Smooth surfaces	• Rigid substrates	
	• Polyimide	• Excellent feature resolution	• Cleanroom requirement	
			• Low scalability	
Soft lithography	• PDMS	• Flexible	• Material permeability	105, 109–112
	• Thermoplastics	• Biocompatible	• Swelling	
		• Rapid prototyping	• Limited chemical compatibility	
		• Relative low cost	• Mechanical deformation	
Subtractive micromachining	• Polymers	• Versatile materials	• Surface roughness	114–118
	• Glass	• Mask-less	• Heat-affected zones	
	• Ceramics	• Rapid prototyping	• Depth uniformity challenges	
	• Metals	• Customizable patterns		
Hot embossing	• Thermoplastics (PMMA, PC, COC)	• High precision	• Requires rigid molds	120, 121
		• Suitable for mass production	• Limited flexibility	
		• Smooth microchannels	• Residual stress	
Additive manufacturing	• Photopolymers	• Highly customizable	• Limited resolution	122–127
	• Hydrogels	• Multi-material integration	• Surface roughness	
	• Bioresorbable polymers	• Rapid prototyping	• Material biocompatibility concerns	
		• Complex geometries	• Slower high-resolution processes	
Fiber-based fabrication methods	• Threads	• Inherent flexibility	• Limited flow control precision	9, 62, 128, 129
	• Paper	• Scalability	• Environmental sensitivity	
	• Textiles	• Low cost	• Challenges in electronic integration	
		• Simple fabrication methods		
		• Natural fluid transport *via* capillary action		

### 3.2. Fabrication strategies for wearable microfluidics

Wearable microfluidic devices require fabrication strategies that ensure structural adaptability, durability, and effective biofluid management while maintaining user comfort. Among the various approaches, polymer-based and fiber-based platforms are widely explored due to their adaptability and compatibility with biosensing technologies. Polymer-based microfluidics offer tunable material properties and precise microchannel fabrication, making them well-suited for wearable biosensors.^8^ Meanwhile, fiber-based microfluidics leverage textiles, threads, and paper substrates to create flexible, breathable platforms for passive biofluid transport.^137^

The following subsections highlight examples of how fabrication techniques have been adapted for polymer-based and fiber-based wearable microfluidic devices, demonstrating their role in real-time health monitoring, personalized diagnostics, and scalable manufacturing.

#### 3.2.1. Polymer-based wearable microfluidics

**Fig. 3:**
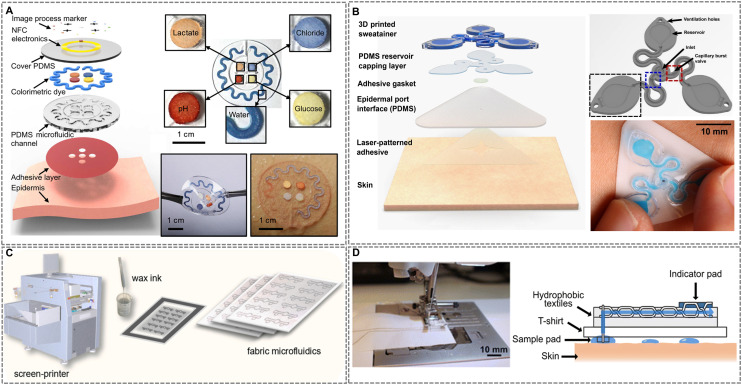
Representative fabrication strategies for wearable microfluidic devices. (A) Soft lithography-enabled multiplexed sweat analysis patch. Adapted with permission from ref. 139, copyright 2016 American Association for the Advancement of Science. (B) 3D printing-enabled “sweatainer” for structured sweat collection. Adapted with permission from ref. 21, copyright 2023 American Association for the Advancement of Science. (C) Wax screen printing-enabled, post-treatment-free fabric microfluidics. Reproduced with permission from ref. 155, copyright 2024 Elsevier. (D) Machine-stitched, textile-based capillary-driven microfluidic device. Adapted with permission from ref. 158, copyright 2025 Royal Society of Chemistry.

Soft lithography remains a cornerstone technique for fabricating polymer-based wearable microfluidics.^138^ A notable example is the PDMS-based device by Koh *et al.* (Fig. 3A), engineered for multiplexed sweat analysis and wireless monitoring.^139^ The system comprises three layers: (i) a skin-adhesive substrate with micromachined sweat inlets, (ii) a soft lithography-molded PDMS layer featuring sealed microchannels and reagent-filled reservoirs for colorimetric detection of biomarkers such as pH, chloride, and glucose, and (iii) a flexible NFC antenna for wireless data transfer. With an effective modulus of ∼0.16 MPa, the device conforms closely to skin mechanics, enabling unobtrusive wear. Finite element analysis further validated its mechanical compliance, showing interface stresses under 30% simulated skin strain remained well below the 20 kPa threshold for tactile perception. As one of the earliest demonstrations of integrated, battery-free, flexible colorimetric microfluidic sensing in a wearable format, this work marks a paradigm shift toward autonomous biofluid analysis and reliable on-body operation—helping define a new direction for epidermal health monitoring systems.

Despite its advantages, soft lithography can be labor-intensive and challenging to scale for mass production.^140^ Recent innovations have sought to evolve this foundational technique by enhancing scalability, precision, and material integration while preserving the intrinsic softness and skin-conformability critical to wearable applications. Advances such as multi-layered PDMS structuring, hybrid lithographic approaches, and microcontact printing have improved microchannel resolution and reproducibility on flexible substrates.^141^ However, conventional soft lithography struggles to achieve high-resolution patterning on non-planar or curved surfaces—a critical requirement for anatomically adaptive wearables. To overcome this, An *et al.* integrated thermoforming with soft lithography, enabling precise molding of curved microchannels.^142^ This hybrid approach facilitated the development of microfluidic contact lens sensors for continuous intraocular pressure monitoring, improving adaptability and performance. Concurrently, Zhang *et al.* introduced a transformative one-step process for fabricating liquid metal (LM)-integrated soft microfluidic sensors.^143^ By combining microchannel patterning with electrochemical LM deposition, their method bypassed traditional photolithography and invasive LM injections. The use of electrochemically functionalized LM stamps ensured precise metal transfer onto PDMS, while interfacial hydrogen bonding reinforced mechanical stability and adhesion. These advancements underscore soft lithography's evolving synergy with next-generation fabrication paradigms, expanding its utility in wearable biosensing and underscoring its adaptability to emerging design and material challenges.

3D printing has emerged as a transformative approach for fabricating wearable microfluidics, enabling customizable, multi-material architectures with embedded biosensing capabilities.^144^ A notable innovation is the 3D-printed “sweatainer” (Fig. 3B), which leverages vat photopolymerization to fabricate enclosed microfluidic channels monolithically, bypassing the multi-step assembly required in traditional PDMS-based systems.^145^ This design supports multi-draw sweat collection for both real-time on-body analysis and offline biomarker quantification, enhancing versatility in fluidic handling. By enabling fully three-dimensional microfluidic architectures and integrated capillary control in a single-step process, this work represents a major advance beyond the planar constraints of conventional soft lithography, enabling the development of autonomous, digitally fabricated fluidic systems. Building on this platform-level progress, Chen *et al.* demonstrated a direct ink writing (DIW)-printed flexible wearable monitor for *in situ* sweat analysis.^146^ Although the device adopts a similar colorimetric sensing strategy, the DIW process removes the need for sacrificial materials and incorporates single-atom catalyst (SAC)-based biosensors through pick-and-place assembly. As a notable improvement, this work achieves high sensitivity and selectivity for real-time monitoring of metabolites such as glucose, lactate, and uric acid, reinforcing 3D printing's growing role in uniting structural customization, material versatility, and functional integration in wearable diagnostics.

Complementing these additive manufacturing techniques, laser-induced graphene (LIG) has emerged as a versatile platform for monolithic microfluidic biosensor fabrication. Garland *et al.* demonstrated this potential by employing CO_2_ laser writing to simultaneously pattern microfluidic channels and porous graphene electrodes directly onto polyimide substrates.^147^ This single-step, maskless process eliminates the need for sacrificial materials or multi-step etching, enabling scalable and cost-effective production of biosensing systems. The resulting sensors are seamlessly integrated with flexible tape-based microfluidics, conforming to the skin for real-time monitoring of biomarkers such as glucose, lactate, and sodium ions. By streamlining fabrication and improving deployment efficiency, this approach advances the accessibility and versatility of wearable diagnostics.

Printed fabrication techniques are gaining traction as scalable, cost-effective alternatives for manufacturing wearable microfluidic systems. These methods utilize direct deposition of conductive or polymer inks to define microfluidic channels, reservoirs, and sensor electrodes on flexible substrates, bypassing traditional cleanroom-dependent processes.^8^ For instance, Vinoth *et al.* developed a photolithography-free workflow for sweat-sensing patches by screen-printing carbon ink masters to mold elastomeric microfluidic layers.^148^ This strategy enabled monolithic integration of sweat sampling channels with screen-printed electrochemical sensors, representing practical improvements in fabrication efficiency and accessibility for scalable epidermal diagnostic platforms. Inkjet printing further expands this paradigm by enabling maskless, precise patterning of functional inks (*e.g.*, conductors, hydrophobic barriers) to architect microfluidic networks.^149^

Hybrid fabrication approaches are increasingly merging printed techniques with established methods such as injection molding and laser structuring to expand functionality and scalability. Makhinia *et al.*, for example, introduced a digitally programmable strategy by combining stereolithography (SLA)-printed microchannels with inkjet-printed hydrophilic coatings to achieve capillary-driven flow control, including stop and delay valves—an advance that enabled autonomous sequencing and integration with screen-printed organic electrochemical transistors (OECTs) for real-time chloride ion sensing.^150^ This represents a transformative step toward fully additive, programmable microfluidic systems. In parallel, Chai *et al.* demonstrated a sustainable fabrication route using injection-molded cellulose acetate (CA) substrates structured *via* CO_2_ laser ablation.^151^ By leveraging the inherent hydrophilicity of CA, the device achieved spontaneous capillary flow without requiring additional surface treatment, supporting functionalities such as droplet generation and passive mixing. While the fabrication methods themselves are established, the use of biodegradable materials and laser-tuned flow behavior marks a strong incremental improvement in eco-conscious microfluidic design. Together, these innovations illustrate how hybrid strategies are advancing microfluidic platforms by integrating digital control, material sustainability, and scalable manufacturing.

#### 3.2.2. Fiber-based wearable microfluidics

Fiber-based microfluidic systems have emerged as a versatile, scalable platform for wearable biosensing, leveraging porous textiles, threads, and paper substrates to enable passive, capillary-driven fluid transport.^152^ Among these, paper-based microfluidics represent a paradigm shift in low-cost, disposable diagnostics.^153^ A prominent approach involves the use of wax-based inks to create hydrophobic barriers on porous fabrics, directing fluid flow through predefined hydrophilic pathways. For example, Cheng *et al.* developed an origami-structured sweat sensor using hydrophilic/hydrophobic filter paper layers folded into programmable microfluidic channels.^154^ By patterning hydrophobic wax barriers *via* laser printing, the device directs sequential sweat flow to integrated colorimetric assays (glucose, lactate, uric acid, pH, and magnesium ions) and electrochemical cortisol sensors. Screen-printed electrodes and synchronized enzymatic reactions with molecularly imprinted polymers (MIPs) enable multiplexed signal capture within a single 30-minute assay. Combined with smartphone-based RGB analysis, this foldable design represents a notable advance in functional integration and usability for mass-producible, point-of-care sweat diagnostics.

Despite its advantages, conventional wax printing often requires post-heating optimization to balance hydrophobic barrier integrity with channel resolution. Addressing this limitation, Tzianni *et al.* (Fig. 3C) developed a wax screen-printable ink for direct, post-treatment-free fabrication of hydrophobic barriers on cotton/elastane fabrics.^155^ This method achieves high-resolution patterning with robust adhesion and reproducibility, critical for high-throughput manufacturing. The resulting screen-printed fabric microfluidic devices (μFADs) integrate colorimetric assays for multiplexed sweat analysis (*e.g.*, pH and urea detection), exemplifying a mass-producible, cost-effective diagnostic platform.

Beyond wax-based methods, xurography offers a rapid, cost-effective alternative for patterning flexible substrates. Kongkaew *et al.* developed a craft-and-stick xurographic method, utilizing a computer-controlled cutting plotter to precisely pattern graphene paper electrodes (GPEs) and polyethylene terephthalate (PET) microfluidic layers.^156^ The components were assembled using adhesive tape, forming a thin, lightweight, and flexible microfluidic-integrated GPE (MF-iGPE). This technique enables rapid prototyping with high reproducibility while eliminating the need for complex lithographic or chemical processes.

Sewing and embroidery techniques have broadened the fabrication toolkit for fiber-based microfluidics by enabling precise integration of hydrophilic threads into textile substrates. Zhao *et al.* demonstrated this with a thread/fabric-based wearable microfluidic device (μTFAD), where hydrophilic threads were embedded within hydrophobic fabric to form directional microchannels for sweat transport.^157^ These channels guided fluid toward colorimetric sensing zones for real-time analysis of pH, chloride, and glucose, with smartphone-based RGB analysis enhancing readout accuracy. The use of automated embroidery supports scalable production while preserving the flexibility and durability required for conformal wear. Expanding on this approach, Hanze *et al.* developed 3D stitched textile microfluidics using computerized embroidery to pattern hydrophilic Coolmax® polyester yarn onto hydrophobic fabric, forming complex 2D/3D channel architectures for capillary-driven fluid mixing and separation (Fig. 3D).^158^ In parallel, gold-coated conductive threads were co-embroidered to function as electrochemical sensors, enabling real-time biomarker monitoring in a reusable, machine-washable T-shirt platform. This method aligns with conventional garment manufacturing workflows, offering a scalable route to discreet, high-performance wearable diagnostics integrated into everyday clothing.

Expanding fabrication versatility, hybrid approaches combine multiple techniques to enhance performance and manufacturability. Li *et al.* developed a plasmonic paper-based microfluidic device integrating laser cutting, hydrophobic patterning, and screen-printed electrodes for surface-enhanced Raman scattering (SERS) sweat sensing.^159^ This device regulates sweat transport through segmented channels, achieving real-time uric acid and pH detection while maintaining structural flexibility and high signal reproducibility. By merging printed electronics, microfluidic patterning, and optical sensing, hybrid fabrication methods optimize precision, scalability, and functionality, further pushing the boundaries of wearable microfluidic biosensing.

To complement these diverse fabrication strategies, it is important to critically assess the translational challenges facing fiber-based microfluidic systems. While paper-based platforms offer distinct advantages—such as low cost, ease of patterning, and capillary-driven flow—their long-term deployment in wearable diagnostics remains limited. Mechanical fragility, sensitivity to environmental fluctuations (*e.g.*, humidity, temperature), and degradation of signal fidelity due to chromogen leaching or enzymatic instability are persistent issues.^160^ These issues extend to textile- and thread-based systems, which, despite improved mechanical flexibility and garment integration, face parallel hurdles. Sweat and skin oils can foul conductive inks or fibers, causing signal drift, while frequent washing and prolonged wear can degrade printed or embroidered electrodes. Additionally, waterproofing and electrical insulation—often *via* laminates or coatings—increase fabrication complexity and may impact recyclability and breathability.^161^ Despite promising prototypes, few fiber-based systems have reached regulatory approval or commercialization. Advancing material durability, standardized encapsulation, and protective coatings will be key to enabling reliable real-world deployment.

### 3.3. Fabrication strategies for implantable microfluidics

The fabrication of implantable microfluidic systems necessitates the integration of high-resolution fluidic channels, biocompatible and often biodegradable materials, and robust yet minimally invasive packaging strategies. These systems must maintain mechanical integrity and functional stability within dynamic biological environments over clinically relevant timescales.^28^ To meet these multifaceted requirements, a diverse set of microfabrication strategies has been explored. This section categorizes key fabrication approaches into molding and sacrificial techniques, lithography-based methods, additive manufacturing, and textile-based integrations, emphasizing their unique advantages, limitations, and representative applications.

#### 3.3.1. Molding and sacrificial assembly techniques

**Fig. 4:**
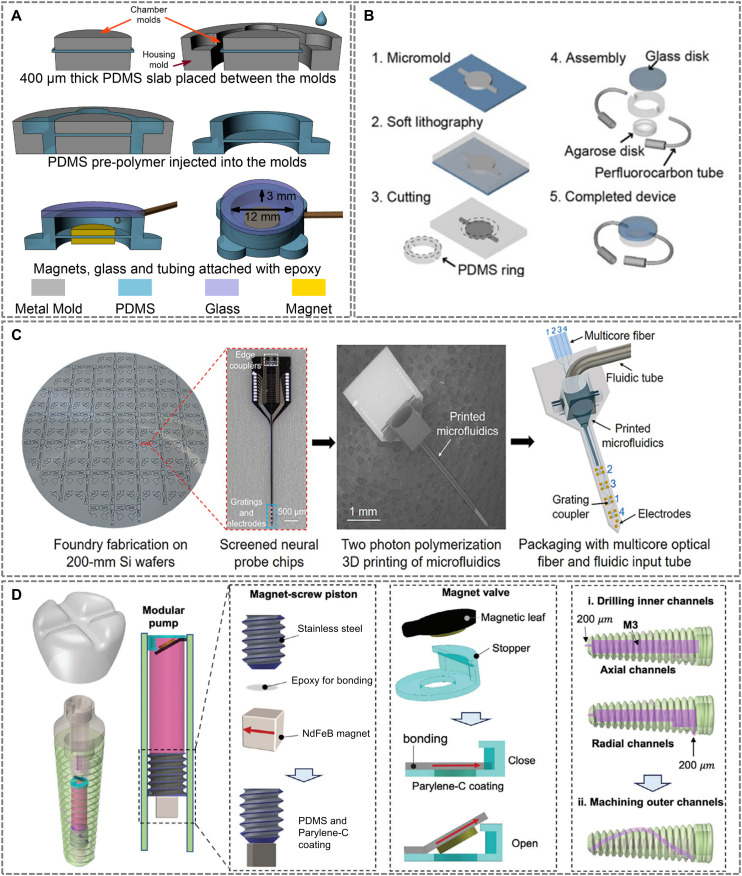
Representative fabrication strategies for implantable microfluidic devices. (A) A magnetic microfluidic pump fabricated *via* PDMS injection molding. Adapted with permission from ref. 162, copyright 2020 MDPI. (B) A PDMS glass microfluidic platform fabricated using micro-molding and soft lithography for neural applications. Adapted with permission from ref. 166, copyright 2014 Springer Nature. (C) Workflow for integrating microfluidic channels with photonic neural probes. Reproduced with permission from ref. 172, copyright 2023 Frontiers. (D) Design of a fluidic-enabled dental implant. Adapted with permission from ref. 173, copyright 2024 Wiley-VCH GmbH.

Molding-based approaches are among the earliest and most versatile methods used to fabricate microfluidic architectures, particularly when high throughput and geometric complexity are required. These techniques utilize rigid molds to shape elastomeric or hydrogel-based materials into functional microfluidic components, often suitable for integration with other soft biointerfaces. For example, Chen *et al.* (Fig. 4A) developed an implantable magnetic microfluidic pump with a diameter of 22 mm and a thickness of 5 mm using polydimethylsiloxane (PDMS) cast within precision-machined stainless-steel molds *via* injection molding to yield a thickness of approximately 400 μm.^162^ Such defined dimensions highlight the capability to achieve high geometric fidelity, which is critical for the intended *in vivo* application in small anatomical spaces like the rat femoral intramedullary cavity.

Sacrificial molding expands the design space by allowing fabrication of enclosed 3D microchannel networks within otherwise difficult-to-pattern matrices. Zhao *et al.* utilized gelatin as a sacrificial template to create microchannels within a silk protein-based hydrogel, resulting in a bioresorbable platform with interconnected vascular-like networks.^163^ This method, achieving channel dimensions as small as 100 μm, is particularly attractive for soft implants intended for tissue regeneration, drug delivery, and other transient applications where biodegradability and biocompatibility are essential.

While conventional molding offers scalable and reproducible fabrication, the use of bioresorbable sacrificial templates like gelatin within biodegradable hydrogels represents a transformative approach. This strategy enables fully transient, tissue-mimicking architectures for regenerative implants—moving beyond static structural replication toward dynamic, biointegrated systems. These molding-based strategies demonstrate a clear advantage in forming complex, bio-integrated 3D microstructures and are often paired with soft materials to match tissue compliance, making them well-suited for long-term implantation.

#### 3.3.2. Lithography-based fabrication techniques

Lithographic techniques, particularly soft lithography, have been instrumental in the evolution of planar microfluidics and continue to be central to the fabrication of high-resolution, functionally integrated implantable devices. These methods offer precise control over feature dimensions and alignment, allowing the creation of microchannels, valves, and hybrid interfaces with sub-micron accuracy.^164,165^ A representative example is shown in Fig. 4B, researchers fabricated a 2.7 mm diameter, 450 μm thick circular window PDMS-glass microfluidic platform for neural applications using soft lithography.^166^ The integration of a fused silica window enabled simultaneous two-photon imaging and localized drug delivery to the mouse cortex, demonstrating the effectiveness of lithographically patterned microchannels for seamless interaction with central nervous system tissues.

To achieve further miniaturization and effective integration with rigid substrates, a thin-film transfer method was employed using dual-depth silicon molds fabricated through deep reactive-ion etching (DRIE).^167^ This approach enabled the one-step fabrication of PDMS channels with a depth of 10 μm, which were subsequently bonded to silicon dioxide surfaces using oxygen plasma. The resulting structure facilitated seamless incorporation of microfluidics onto silicon-based neural probes, supporting precise nanoliter-scale drug delivery.

Expanding on the versatility of lithographic methods, researchers have demonstrated the use of multilayer lithography for the integration of optical and fluidic functionalities.^168^ They developed a fully implantable optofluidic cuff by employing adhesive and plasma bonding techniques for accurate multilayer alignment, achieving interlayer registration within ∼50 μm. This configuration allowed for targeted fluid delivery through the microfluidic channels with cross-sectional areas of 60 × 60 μm alongside neural interfacing capabilities, illustrating the modularity and multifunctional potential of layered lithographic fabrication for complex implantable systems.

To improve long-term device stability and prevent gas permeation, a parylene-coated microfluidic system was developed and embedded within an intraocular lens.^169^ The PDMS microchannels were fabricated using standard soft lithography techniques, achieving feature resolutions down to 50 μm. The channels had dimensions of 50 × 50 μm^2^ in cross-section and lengths on the order of several millimeters, connected to a gas reservoir measuring approximately 500 × 500 × 300 μm^3^. A thin, uniform parylene-C coating was deposited onto the PDMS structures to significantly reduce gas permeability. This approach was particularly advantageous for preserving delicate microstructures in sensitive ophthalmic environments, ensuring durability and functional integrity over extended periods of implantation.

A more radical implementation of lithography-enabled microfluidics was presented recently with a soft, bioresorbable evaporative cooling device for nerve modulation.^170^ The fabrication involved poly(octanediol citrate) (POC) as a bioresorbable elastomer to create microchannels through soft lithography techniques, achieving serpentine geometries with precisely controlled dimensions and a cuff structure tailored specifically to a 1.5 mm nerve diameter. The device integrated bioresorbable magnesium-based temperature sensors patterned onto cellulose acetate substrates, facilitating real-time temperature feedback. The microfluidic channels, with widths and lengths optimized to about 100 μm and several millimeters respectively, successfully delivered perfluoropentane and nitrogen, enabling localized evaporative cooling without the need for sutures or explantation. This illustrates the remarkable potential of lithographic methods in transient therapeutic systems requiring precise spatiotemporal control.

While many of the fabrication techniques discussed—such as soft lithography or micromolding—offer incremental refinements in biocompatibility and resolution, certain approaches represent more transformative shifts. Notably, hybrid lithography strategies combining soft and hard lithography for multilayer optofluidic integration enable unprecedented neural interfacing capabilities. Similarly, the use of bioresorbable materials patterned *via* photolithography to create transient, stitch-free evaporative cooling devices signifies a paradigm shift in temporary implant design. These advances go beyond performance optimization, fundamentally altering how microfluidic systems interface with biological tissue and resolve long-standing challenges in surgical retrieval, device miniaturization, and multimodal function.

#### 3.3.3. Additive manufacturing and printed microfluidics

Additive manufacturing (AM) has introduced a paradigm shift in microfluidics by enabling rapid, on-demand fabrication of complex, three-dimensional architectures on both flat and irregular substrates. Techniques such as stereolithography (SLA), two-photon polymerization (2PP), and aerosol-jet printing allow for the integration of microfluidics with electronics, optics, and soft robotics in a compact form factor.

Ives *et al.* designed a microfluidic force sensor embedded within a 3D-printed hip implant using SLA for the fluidic structure and aerosol-jet printing for flexible electrodes on Kapton.^171^ The device withstood surgical loads exceeding 400 N and maintained a compact, mechanically adaptive profile. Bonding was achieved using precision-cut double-sided adhesives, demonstrating the feasibility of AM techniques for orthopedic applications requiring structural durability.

At the microscale, Mu *et al.* (Fig. 4C) used 2PP to fabricate fluidic channels directly onto photonic neural probes, achieving nanoscale alignment between waveguides, electrodes, and microchannels.^172^ Printed using a high-resolution Nanoscribe system and IP-S resin, the integrated structure exhibited precise dimensions, with channel inner dimensions of approximately 70 μm width and 18 μm height, highlighting the high fabrication precision attainable with 2PP. These probes enabled localized, addressable uncaging of fluorescein at micrometer-scale resolution, demonstrating the capability of 2PP for achieving not only structural precision but also functional precision through highly controlled fluid and light delivery. Furthermore, Mu *et al.* confirmed strong adhesion and structural integrity of the printed microfluidics, even after repeated insertions into brain tissue, underscoring the excellent mechanical stability and surface quality achievable *via* this technique. This approach underscores the strength of 2PP in achieving seamless optofluidic integration for high-precision neuromodulation.

A distinctive example of functional integration using AM is provided by Xu *et al.* (Fig. 4D), who engineered a wirelessly actuated pump and valve system within a dental implant.^173^ The system utilized an SLA-printed body, a PDMS- and parylene-C-coated steel piston with an embedded magnet, and a magnetically actuated Ecoflex valve. Wireless operation was achieved *via* an external rotating magnetic field, enabling localized, on-demand therapeutic delivery to the bone–implant interface. This comprehensive design exemplifies the synergy between 3D printing, soft magnetics, and remote actuation for smart therapeutic implants.

#### 3.3.4. Textile-based integration techniques

Textile-inspired microfluidics offer an unconventional yet highly effective approach for embedding sensing and fluidic functionality into biologically compliant, soft materials. Threads and yarns inherently possess properties such as flexibility, porosity, and capillarity, which can be harnessed to form microfluidic networks and biosensors that conform seamlessly to biological tissues.

Thread-based microfluidics were pioneered through the sequential coating of cotton threads with conductive and functional inks to create flexible, integrated fluidic systems.^174^ Mostafalu *et al.* employed dip-coating processes to functionalize threads with carbon nanotubes (CNTs), polyaniline (PANI), and silver/silver chloride inks, yielding conductive threads with microscale features governed primarily by thread diameter (ranging from tens to hundreds of micrometers). SEM analysis indicated that functional nanomaterials effectively infiltrated thread pores, creating stable, interconnected conductive surfaces suitable for reliable electrochemical sensing. Each coated layer was carefully stabilized through drying and curing steps to enhance mechanical integrity and prevent delamination under strain. The resulting robust, flexible sensors demonstrated high sensitivity, with strain sensors achieving gauge factors around 3 at strains up to 100%, while chemical sensors maintained stable, near-Nernstian responses (−59.63 mV pH^−1^) with minimal drift (2.5 mV h^−1^). These thread-based platforms, with their precise and scalable fabrication, can be directly embedded into biological tissues, offering a minimally invasive route for continuous *in situ* biochemical monitoring and therapeutic actuation within soft, tissue-compliant environments.

While thread-based platforms originated as low-cost flexible sensors, their evolution into multi-layered, tissue-compatible sensing and drug delivery systems suggests a transformative direction. These platforms challenge traditional rigid implant geometries by embedding capillary networks and electronics within soft, thread-like substrates that mimic native tissue architecture.

## 4. Applications of microfluidic devices

The previous sections have outlined the fundamental design principles and fabrication strategies that underpin the development of wearable and implantable microfluidic systems. These innovations have paved the way for practical biomedical applications, where microfluidic devices serve as powerful tools for continuous health monitoring, targeted drug delivery, and real-time biochemical analysis. This section focuses on the practical applications of microfluidic technology in both wearable and implantable formats. Wearable microfluidic systems have been developed for non-invasive health monitoring through sweat analysis, epidermal biosensing, and integration into smart textiles, offering continuous and personalized diagnostics in real-time. In contrast, implantable microfluidic platforms are engineered for *in vivo* monitoring, precise drug delivery, and interfacing with neural and biochemical environments, enabling long-term therapeutic interventions with minimal patient discomfort. Together, these innovations underscore the transformative impact of microfluidics in advancing minimally invasive, responsive, and patient-centric healthcare solutions.

### 4.1. Applications of wearable microfluidic devices

Wearable microfluidic devices represent a major advancement in biomedical sensing, enabling non-invasive, continuous, and personalized health monitoring through real-time analysis of biofluids. By integrating soft, skin-conformal materials with passive or low-power fluid manipulation strategies, these systems achieve long-term compatibility with the human body. The following subsections examine representative devices by targeted biofluid. Table 3 presents a comparative summary of recent progress across sweat, tears, interstitial fluid, wound exudate, saliva, and genitourinary secretions—highlighting sensing targets, detection methods, application areas, clinical readiness, and remaining challenges.

**Table 3:** Overview of wearable microfluidic platforms across biofluids: targets, applications, detection strategies, and translational status

Biofluid	Sensing analytes	Key features	Clinical status	Key challenges	Ref.
Sweat	Na^+^, K^+^, Cl^−^, glucose, lactate, cortisol, alcohol, metabolic biomarkers	Skin-mounted patches/smart textiles with microfluidic design embedded with electrochemical or colorimetric sensors	Commercial for hydration (*e.g.* Gx patch), most in preclinical stage	Sweat rate variability, evaporation, analyte calibration, sample collection under rest condition	21, 175–199
Interstitial fluid (ISF)	Glucose, lactate, ethanol, therapeutic drugs	Microneedle arrays for continuous ISF access; stretchable microfluidic-microneedle platforms	Early-stage clinical validation	Minimally invasive insertion, sensor biofouling, calibration accuracy, extraction efficiency, regulatory approval	200–205
Saliva	Cortisol, glucose, lactate, IgA, uric acid, bacteria	Wearable mouthguards and tooth-mounted sensors; hydration-sensitive microfluidic designs	Prototypes and limited human trials	Food contamination, variable composition, device comfort and sensor stability	206–212
Tears	Glucose, lactate, electrolytes, proteins, IOP, cytokines	Smart contact lens sensors for multiplexed tear biomarker sensing; strain sensitive optofluidic designs	Early-stage clinical validation	Micro-volume handling, biocompatibility, optical clarity, data transmission	213–224
Wound exudate	pH, ROS (NO, H_2_O_2_), cytokines, bacteria, temperature, moisture	Smart dressings with passive wicking; printed colorimetric/electrochemical biosensors; real time feed back to care givers	Animal studies, limited human pilots	Sensor fouling, low exudate in chronic wounds, wireless communication and power in disposable forms	225–231
Genitourinary (urine; vaginal fluid)	Electrolytes, glucose, creatinine, nitrite, protein, pH	Smart diapers with multi-ion detection, integrated wireless system; vaginal ring biosensors; sanitary pad embedded sensors	Prototypes; pre-clinical evaluation	Intermittent flow, sensor saturation, hygiene/infection control, use comfort and private data handling	232–235

#### 4.1.1. Sweat sensors

Sweat-based biosensing has garnered significant interest as a non-invasive, continuous diagnostic modality, offering a rich matrix of biomarkers accessible directly from the skin surface.^175^ Secreted through eccrine glands and available without puncturing the skin, sweat provides analytes including electrolytes (Na^+^, K^+^, Cl^−^), metabolites (lactate, glucose, uric acid), hormones (*e.g.*, cortisol), proteins, and drug residues—all of which reflect hydration status, metabolic function, stress response, and disease progression.^176^ The inherently continuous nature of sweat secretion renders it particularly well-suited for dynamic, real-time health monitoring.^177–181^

However, the practical deployment of sweat sensors faces several biochemical and engineering challenges. Sweat analyte concentrations are typically lower than those found in blood or interstitial fluid, necessitating sensitive detection schemes. Furthermore, sample volume is limited and highly variable, with environmental factors (*e.g.*, temperature, humidity) and individual physiological conditions (*e.g.*, gland density, physical activity) affecting both sweat rate and analyte composition.^177^ Rapid evaporation and potential contamination from skin surfaces also threaten sample fidelity. Microfluidic technologies mitigate many of these limitations by enabling precise routing, collection, and isolation of minute sweat volumes while facilitating controlled delivery to embedded sensors.^21,182,183^

**Fig. 5:**
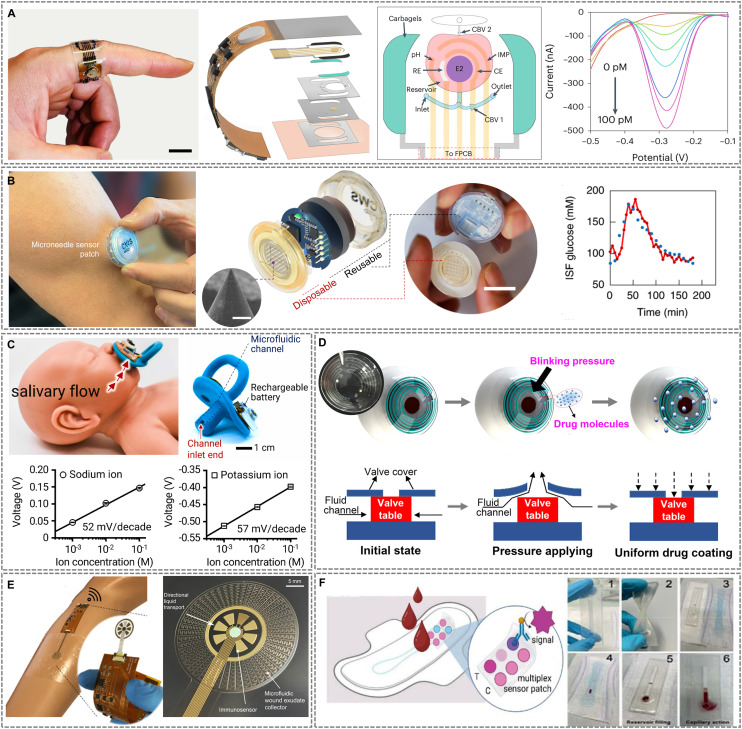
Representative applications of wearable microfluidic devices for biofluid sensing and therapeutic functions. (A) Aptamer-based electrochemical sweat sensor for estradiol detection. Adapted with permission from ref. 191, copyright 2023 Springer Nature. (B) Microneedle-enabled ISF biosensor for multiplexed metabolite monitoring. Adapted with permission from ref. 205, copyright 2022 Springer Nature. (C) Smart bioelectronic pacifier for salivary ion monitoring in neonates. Reproduced with permission from ref. 212, copyright 2022 Elsevier. (D) Pressure-actuated microfluidic contact lens for ocular drug delivery. Adapted with permission from ref. 225, copyright 2022 American Chemical Society. (E) Wound-exudate microfluidic patch with bioinspired fluidic collector. Adapted with permission from ref. 231, copyright 2021 American Association for the Advancement of Science. (F) In-pad diagnostic platform for multiplexed menstrual biomarker detection. (1 and 2) Soft-silicon casing for embedding paper-based sensors. (3) Placement of the device into a sanitary pad. (4) Blood collection on the first fluid transfer layer. (5 and 6) Capillary-based intake of a controlled volume and completion of the LFA test. Adapted with permission from ref. 236, copyright 2025 Wiley-VCH GmbH.

Recent developments illustrate how integrated microfluidic architectures can enhance analytical performance while maintaining device wearability and user comfort.^184–190^ Ye *et al.* designed a fully autonomous aptamer-based biosensor for estradiol monitoring in sweat (Fig. 5A).^191^ The device employed a reagentless “signal-on” detection strategy based on strand displacement, coupled with microfluidic iontophoresis for controlled sweat stimulation and sample acquisition. Embedded sensors for pH, ionic strength, and temperature enabled real-time calibration, while gold nanoparticle–MXene electrodes enhanced electrochemical sensitivity, achieving an ultralow detection limit of 0.14 pM and excellent batch-to-batch reproducibility. The system demonstrated strong correlation with serum hormone levels in clinical studies, highlighting its potential for real-time, at-home monitoring of reproductive health.

Beyond hormonal monitoring, the functional scope of sweat sensors has expanded to include metabolic profiling and pharmacological tracking.^192–194^ Cho *et al.* introduced a battery-free, fluorometric enzymatic patch that combined time-sequenced reservoirs and passive microvalves within a PDMS platform.^195^ Smartphone-assisted imaging enabled multiplexed detection of amino acids such as lysine, with a detection limit of 0.13 μM, offering insights into exercise-induced amino acid loss and guiding nutritional supplementation strategies. Complementing this metabolic application, Xiao *et al.* developed a flexible surface-enhanced Raman spectroscopy (SERS)-based patch for acetaminophen detection.^196^ The device incorporated an Au nanosphere cone array within a skin-conformal microfluidic chip, enabling label-free detection of acetaminophen down to 0.13 μM. Real-time drug metabolism profiles were captured in human subjects using a portable Raman spectrometer, with results showing strong agreement with HPLC analysis, underscoring the platform's translational potential for noninvasive, personalized drug monitoring.

Recent efforts have focused on leveraging localized sweat analysis to extract physiological insights.^197,198^ A skin-interfaced microfluidic band developed by Cho *et al.* enabled time-resolved mapping of sweat biochemistry across body locations and exercise conditions, incorporating a colorimetric timing module to track dynamic changes in pH and lactate during physical activity.^199^ The study demonstrated a strong correlation between sweat pH and blood lactate over active muscle groups, establishing sweat pH as a viable non-invasive biomarker for muscle fatigue and exertion levels. Together, these studies exemplify how skin-conformal sweat microfluidic sensors can support both performance optimization and individualized metabolic health monitoring in real-world settings.

#### 4.1.2. ISF sensors

Interstitial fluid (ISF) has emerged as a compelling alternative to blood for wearable biosensing due to its compositional similarity and minimally invasive accessibility. As the extracellular fluid surrounding tissue cells, ISF serves as a critical medium for nutrient exchange and metabolic signaling.^200^ Importantly, concentrations of key biomarkers—such as glucose, lactate, electrolytes, and therapeutic drugs—closely parallel those found in blood, making ISF an attractive candidate for continuous health monitoring without the need for venipuncture or implantable vascular access.^201^

Although ISF holds strong potential for wearable biosensing, physiological constraints present challenges. While the dermal layer contains a substantial ISF reservoir, only a small amount is accessible at the surface due to transport resistance and slow replenishment from the bloodstream. Additionally, repeated or prolonged extraction poses risks of tissue irritation or fibrosis. As such, wearable ISF sensors must be engineered to maximize analytical yield from minimal sample volumes while maintaining mechanical compliance and user comfort.^202^

Microfluidic integration offers clear advantages in this context by facilitating efficient collection, routing, and analysis of trace fluids, while maintaining strict spatial confinement to avoid contamination and evaporation. Recent innovations have demonstrated the feasibility of microneedle-integrated microfluidic systems that support multiplexed biosensing with high sensitivity.^203^

One notable example is the microneedle-based microfluidic patch developed by Silva *et al.*, which utilizes two-photon polymerization to fabricate hollow microneedles directly onto microfluidic substrates.^204^ The needles demonstrated mechanical strength of 411 ± 3 mN per needle, sufficient to withstand at least 10 consecutive insertions into skin-mimicking materials without deformation. *In vivo* evaluations conducted over a 72-hour period confirmed the device's biocompatibility, structural reliability, and capability for fast and consistent ISF extraction. This approach highlights the growing role of advanced microfabrication and integration strategies in enhancing ISF accessibility and reliability for continuous biomarker sensing.

Another notable example is the fully integrated wearable microneedle sensor developed by Tehrani *et al.* (Fig. 5B), which enables real-time, continuous monitoring of glucose, lactate, and alcohol in ISF.^205^ The system employs a microneedle array manufactured *via* a high-resolution CNC micromachining process, allowing precise fabrication of high-aspect-ratio channels and robust through-holes for effective ISF extraction and guided transport. Multiplexed electrochemical sensors and low-power custom electronics are embedded to support wireless data transmission and app-based visualization. On-body trials confirmed a glucose detection limit of 0.32 mM and a mean absolute relative difference (MARD) of 9.6% compared to standard blood glucose meters. Dual-analyte sensing with minimal crosstalk was achieved through spatial electrode separation and tailored sensing chemistry. These advances reflect the rapid maturation of ISF biosensing platforms, transitioning from invasive subdermal systems toward epidermal patches capable of autonomous, continuous monitoring.

#### 4.1.3. Saliva sensors

Saliva plays a vital role in maintaining oral and systemic health by lubricating the mouth, aiding digestion, protecting teeth from decay, and providing antimicrobial defense. It has also gained attention as a biofluid for wearable sensing due to its non-invasive, stress-free collection and rich molecular composition.^206^ Secreted primarily by the parotid, submandibular, and sublingual glands, saliva contains diverse analytes—including glucose, cortisol, cytokines, immunoglobulins (*e.g.*, IgA), enzymes (*e.g.*, amylase), C-reactive protein (CRP), and drug metabolites—many of which reflect systemic conditions ranging from metabolic and inflammatory diseases to viral infections and psychological stress.^207^ The ease of access and continuous secretion of saliva make it suitable for continuous monitoring in both clinical and home settings.^208,209^

However, the dynamic nature of the oral environment presents substantial engineering and analytical challenges. Salivary flow rates can vary significantly with hydration status, circadian rhythms, and user activity (*e.g.*, eating, speaking), leading to signal variability and inconsistent sample volumes. Moreover, low analyte concentrations, enzymatic degradation, and interference from oral microbiota complicate both sample stability and sensor performance.^210^ These factors necessitate robust sample conditioning, precise fluid handling, and high analytical sensitivity within wearable form factors designed for intraoral or perioral integration. Microfluidic systems offer a compelling solution by enabling precise, small-volume sample routing, built-in filtration, and real-time analysis in compact, skin- or mouth-mounted formats. These devices can be seamlessly integrated with biocompatible polymers, wireless communication modules, and low-power sensors to create closed-loop platforms suitable for both clinical and home-based use.^211^

An illustrative example is the smart bioelectronic pacifier by Lim *et al.* (Fig. 5C), designed for real-time monitoring of salivary sodium and potassium in neonates.^212^ The system embeds solid-state ion-selective electrodes (SS-ISEs), a PDMS-PEG microfluidic channel, and a wireless circuit into a compact pacifier form. Capillary-driven flow enables passive, continuous sampling without the need for suction or user interaction. The sensors exhibit high sensitivity—53 mV per decade for sodium and 63 mV per decade for potassium—and stable output over 10 hours. *In vivo* testing showed reliable tracking of salivary ion levels (sodium: 5.7–9.1 mM; potassium: 4.2–5.2 mM), demonstrating the system's suitability for non-invasive, neonatal electrolyte monitoring.

Another example is the wearable mouthguard sensor developed by de Castro *et al.*, which integrates microfluidic paper-based analytical devices (μPADs) for salivary glucose and nitrite detection.^213^ Fabricated *via* craft cutter printing, the μPADs feature two detection zones with colorimetric readouts and achieved low detection limits of 27 μmol L^−1^ for glucose and 7 μmol L^−1^ for nitrite. In clinical samples, the device reliably distinguished elevated glucose and nitrite levels in patients with diabetes and periodontitis, respectively. Its low cost, simplicity, and instrument-free operation make it a promising platform for accessible salivary diagnostics.

#### 4.1.4. Tear sensors

Tears are a complex biological fluid secreted primarily by the lacrimal glands, with contributions from conjunctival goblet and accessory glands. They serve essential physiological functions—lubricating the ocular surface, maintaining corneal integrity, and providing antimicrobial defense. Beyond their protective role, tears are increasingly recognized as a valuable diagnostic medium, as they contain a variety of biomarkers such as glucose, lactate, electrolytes (*e.g.*, sodium, potassium), proteins, enzymes, lipids, hormones, and drug metabolites. Due to the blood–tear barrier, many of these analytes closely correlate with blood concentrations, making tears a minimally invasive proxy for systemic monitoring. These features render tear-based sensing particularly attractive for the detection and monitoring of metabolic, ocular, and neurodegenerative disorders.^214,215^

Wearable tear sensors have evolved across several platforms, including electrochemical patches near the eye and, more prominently, smart contact lenses.^216^ These lenses offer a unique advantage by resting directly in contact with the tear film, enabling continuous, real-time monitoring of tear fluid composition. Among the targeted analytes, glucose is the most widely explored, especially for diabetes management.^217^ Other indicators include intraocular pressure (IOP), pH, lactate, electrolytes, and therapeutic drug levels.^218^ Microfluidic contact lenses (MCLs) stand out in this landscape for their ability to precisely manipulate ultra-small tear volumes within integrated channel networks.^219^ They support localized reagent mixing, improve analyte detection efficiency, and minimize contamination—benefits critical to both biosensing and ocular drug delivery.^220^

A representative example is the AI-assisted wearable microfluidic colorimetric sensor (AI-WMCS) introduced by Wang *et al.*, which enables multi-analyte detection in tear fluid using smartphone-based image analysis.^221^ The PDMS-based microfluidic patch detects vitamin C, pH, calcium ions, and proteins with minimal sample volume (∼20 μL), and integrates a cloud-based deep learning algorithm to automatically correct for ambient lighting and pH-induced signal variation. The system achieved excellent analytical performance (*R*^2^ > 0.99 across targets), highlighting its potential for remote diagnostics and telehealth.

Building on the eye-conformal sensing paradigm, Shi *et al.* developed a fluorescent smart contact lens for real-time detection of glutathione (GSH), a key oxidative stress biomarker.^222^ The device incorporates a coumarin-based probe within a laser-patterned microfluidic channel to autonomously collect tear fluid and transduce GSH concentrations through a reversible Michael addition reaction. Smartphone-assisted hue analysis allowed detection of physiological GSH levels (0.15–1.05 mM) with a sensitivity of 0.12 mM, and week-long stability testing confirmed biocompatibility for extended use.

Further expanding this class of diagnostic lenses, Moreddu *et al.* demonstrated a microfluidic contact lens integrating paper-based colorimetric sensors for simultaneous detection of five tear biomarkers, including glucose, pH, proteins, nitrites, and l-ascorbic acid.^223^ The poly(HEMA) lens design incorporates laser-inscribed microchannels to direct tear fluid (∼2 μL) toward embedded sensing zones, achieving sub-minute response times and clinically relevant detection limits (*e.g.*, 1.1 mmol L^−1^ for glucose). A smartphone app supports real-time image capture and analysis, underscoring the practicality of this platform for point-of-care ocular diagnostics.

Beyond diagnostics, microfluidic contact lenses also offer a promising avenue for ocular drug delivery.^224^ Du *et al.* developed a pressure-triggered microfluidic contact lens that enables controlled release of ophthalmic drugs using a blink-driven micropump system (Fig. 5D).^225^ Unlike traditional eye drops, which suffer from poor retention and low bioavailability, this lens incorporates an embedded microchannel network and a check valve that activates drug release in response to natural eyelid pressure. The device demonstrated the capability to store and release ∼3.5 μL of liquid from compartmentalized drug reservoirs, supporting both small- and large-molecule therapeutics. The PDMS-based design maintains lens comfort and oxygen permeability, establishing a soft and self-actuating drug delivery interface for chronic eye conditions. Collectively, tear-based microfluidic sensors and drug delivery systems represent a convergence of precision engineering, biocompatible materials, and user-centric design.

#### 4.1.5. Wound exudate sensors

Wound exudate is a biologically rich fluid secreted in response to skin injury and plays a vital role in the healing process. It contains a dynamic mixture of water, electrolytes, proteins, immune mediators, and cellular debris that contribute to maintaining moisture, clearing pathogens, and delivering key factors for tissue regeneration. Because its molecular composition evolves with the healing stage, wound exudate provides valuable biochemical cues about the wound's condition. Analyzing components such as cytokines, pH, and enzymes can offer insights into inflammation, infection, and repair status—enabling clinicians to better assess wound severity and guide treatment decisions.^226,227^

However, current clinical wound assessment remains largely qualitative, relying on visual inspection and intermittent sampling, which may delay detection of pathological deterioration, especially in chronic wounds.^228^ Wearable microfluidic platforms offer an elegant solution to these limitations by enabling continuous, real-time monitoring of exudate biochemistry *in situ*. Their soft, flexible architectures conform to irregular wound geometries, and their integrated microchannels allow spatially resolved fluid transport, multiplexed sensing, and even localized therapy.^229^ These systems are often coupled with wireless data acquisition and AI-assisted analytics, transforming wound care from reactive to responsive.^230^

A landmark development is the VeCare platform introduced by Gao *et al.*, which integrates an immunosensor array with a biomimetic microfluidic collector inspired by the Texas horned lizard's skin (Fig. 5E).^231^ The sawtooth-shaped capillary channels enable unidirectional flow of exudate, preventing reverse contamination and ensuring efficient delivery to sensing zones. The device enables multiplexed electrochemical detection of cytokines (TNF-α, IL-6, IL-8, TGF-β1), pH, temperature, and bacterial load (*S. aureus*). Wireless data transmission to a smartphone interface supports remote wound evaluation, and *in vivo* validation in both animal and clinical settings demonstrated high biocompatibility and responsiveness to wound state changes.

In another work, Zheng *et al.* developed the PETAL sensor—a battery-free, paper-based wound sensing platform integrating five colorimetric sensors for temperature, pH, moisture, uric acid, and trimethylamine (TMA).^25^ Fabricated using wax-printed microfluidics, the system incorporates blood filtration and passive sampling layers to manage heterogeneous exudate. Smartphone-based imaging, combined with deep learning algorithms, enables automated classification of wound healing status with up to 97% accuracy. This low-cost, scalable design represents a major step toward low-cost, point-of-care wound diagnostics and early infection warning systems.

To enhance sensitivity and analytical resolution, Chen *et al.* introduced a dual-mode microfluidic chip combining electrochemical and surface-enhanced Raman spectroscopy (SERS) sensing for interleukin-6 (IL-6), a critical inflammatory cytokine.^232^ A square-wave micromixer improves antigen–antibody interaction kinetics, and outputs from both detection modes are fused using a neural network for high-fidelity quantification across a wide dynamic range (0.05–1000 pg mL^−1^). The system achieved detection limits as low as 0.047 pg mL^−1^ (SERS) and 0.085 pg mL^−1^ (EC), validated against ELISA using diabetic wound fluid. Such hybrid platforms enable granular profiling of wound inflammation, enabling proactive, data-driven interventions.

#### 4.1.6. Genitourinary fluid sensors

Genitourinary fluids—including urine, vaginal secretions, and menstrual blood—represent clinically rich yet underutilized media for wearable biosensing. Secreted naturally through the urinary and reproductive tracts, these fluids offer a non-invasive interface for accessing a wide range of biochemical markers linked to local infections, systemic disorders, reproductive health, and hormonal status.^233–236^

Among these fluids, urine remains the most extensively studied in clinical diagnostics due to its abundant volume and diagnostic versatility. It is widely used for detecting kidney diseases, urinary tract infections (UTIs), metabolic disorders, and monitoring hydration status.^237^ Vaginal secretion and menstrual blood are increasingly recognized in clinical research for its potential to reflect bacterial vaginosis, endometrial health, hormonal fluctuations, iron deficiency, and markers associated with reproductive disorders and inflammatory conditions.^235,238^ Their external accessibility and diagnostic relevance make genitourinary fluids ideal targets for wearable sensing technologies, although research in this area is still in its early stages.

Despite their promise, engineering challenges persist. Secretion frequency, sample volume, and biofluid composition can vary widely across individuals and timepoints. Sensor integration must account for fluid intermittency, environmental exposure, and user comfort—particularly for long-term wear in intimate anatomical regions. Furthermore, issues of hygiene, biocompatibility, and privacy require sensitive design strategies to ensure user acceptance.^239^

One recent advance comes from Bi *et al.*, who introduced a universal fully integrated wearable sensor array (FIWSA) designed for simultaneous, noninvasive monitoring of electrolytes (Na^+^, pH) and metabolites (uric acid) across multiple raw biofluids—including sweat, saliva, and urine.^240^ The system combines 3D carbon-based electrochemical sensors, microfluidic routing, and wireless telemetry within a single platform. In urine samples, it enabled accurate monitoring of sodium, pH, and uric acid levels, demonstrating utility for metabolic assessment and hydration tracking. While the proof-of-concept was validated in controlled environments, further optimization for *in situ* urine capture—such as in smart diapers or wearable liners—remains a key translational step.

Expanding to women's health, Dosnon *et al.* introduced MenstruAI, an in-pad diagnostic platform for multiplexed biomarker detection in menstrual blood (Fig. 5F).^236^ The multilayered microfluidic architecture incorporates plasma filtration and lateral flow immunoassay zones to detect markers such as C-reactive protein (CRP), carcinoembryonic antigen (CEA), and CA-125—relevant to inflammation, cancer, and endometriosis, respectively. The platform enables semi-quantitative readouts *via* naked-eye observation or smartphone imaging, with machine learning–assisted interpretation. This work demonstrates the feasibility of non-invasive, self-administered diagnostics for reproductive health and disease screening directly within menstrual hygiene products. Together, these advances highlight the transformative potential of genitourinary fluid microfluidics.

### 4.2. Applications of implantable microfluidic devices

Implantable microfluidic devices are opening new avenues in precision medicine by enabling *in vivo* fluid handling, sensing, and therapeutic delivery at the microscale. These miniaturized systems offer the potential for continuous physiological monitoring and localized treatment, enhancing targeting precision while improving patient compliance. In the following section, we examine representative applications across major biomedical domains, outlining key device types, clinical or research status, and notable studies. Table 4 provides a consolidated overview of emerging implantable microfluidic systems and their translational progress.

**Table 4:** Emerging implantable microfluidic systems for therapeutic delivery, biosensing, and organ support: device features, clinical translation, and challenge

Application	Representative devices	Key features	Clinical status	Challenges	Ref.
Targeted therapeutic delivery	Wireless microchip drug implants, magnetically actuated pumps	• Magnetic actuation, osmotic pressure, electrochemical, TENG, thermal burst	Prototype to early clinical (*e.g.*, TAR-200 in trials)	Powering, refilling, long-term stability, FDA regulation	31, 251–255
		• Target organs: bone, dental, brain, bladder, eye, GI tract			
Artificial organs & organ support	Bioartificial kidney, implantable liver support	• Physiological filtration, solute clearance, liver/pancreatic tissue support, mammary gland genetic fluid delivery	Preclinical to prototype (ongoing kidney/liver support studies)	Immune response, material fouling, energy demands	95, 258–270
		• Target organs: kidney liver, pancreas, mammary glands			
Neural interfaces & neuromodulation	Wireless optofluidic probes, chemtronic neural interfaces, bioresorbable devices	• Optical/electrical/pharmacological control; real-time neural modulation, soft robotics	Mostly preclinical (*in vivo* animal studies)	Miniaturization, biocompatibility, data synchronization	166, 254, 271–280
		• Target organs: brain, spinal cord, peripheral nerves			
Implantable biosensors & diagnostics	Intra-abdominal pressure sensors, force sensors in hip implants	• Continuous pressure'/metabolic sensing; surgical feedback integration	Preclinical (animal models, early surgical deployment)	Calibration drift, encapsulation, wireless data transmission	281–290
		• Target organs: eye, abdomen, joints, brain			
Tissue engineering & regenerative medicine	NIR-responsive VEGF hydrogel scaffolds, microvascularized AngioChip	• Stimulus-responsive scaffolds; vascular integration; cell guidance, bone remodelling	Preclinical (mouse/rabbit models), some human-compatible platforms	Nutrient perfusion, biodegradability, structural durability	162, 174, 291–302
		• Target organs: bone, skin, cartilage			

#### 4.2.1. Targeted therapeutic delivery systems

The targeted delivery of therapeutics using implantable microfluidic systems represents a major advancement in precision medicine. Traditional systemic drug administration often suffers from off-target effects, limited local bioavailability, and poor temporal control.^241–243^ In contrast, implantable microfluidic devices enable spatially and temporally controlled delivery of therapeutic agents directly to the site of interest, minimizing systemic exposure and maximizing treatment efficacy.^244^

**Fig. 6:**
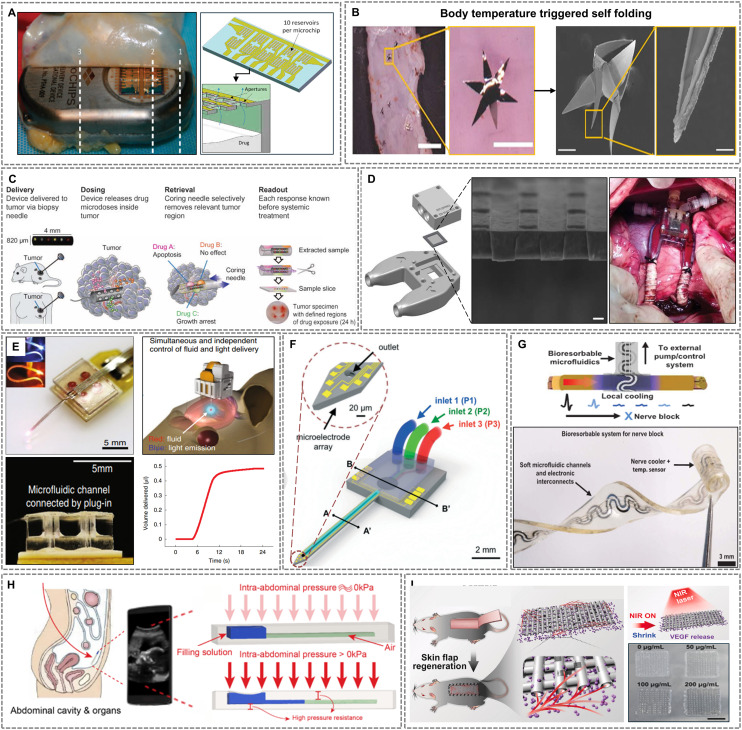
Representative implantable microfluidic systems for therapeutic delivery, neural modulation, organ support, and real-time monitoring. (A) Wireless microchip drug delivery implant for controlled parathyroid hormone release in osteoporosis patients. Adapted with permission from ref. 245, copyright 2012 American Association for the Advancement of Science. (B) Self-folding microinjectors for minimally invasive insulin delivery to the gastrointestinal tract. Adapted with permission from ref. 248, copyright 2022 American Chemical Society. (C) Multiplexed intratumoral microdevice for personalized cancer drug screening. Reproduced with permission from ref. 251, copyright 2015 American Association for the Advancement of Science. (D) Artificial kidney-on-chip integrating glomerular filtration and tubular reabsorption for renal replacement therapy. Adapted with permission from ref. 260, copyright 2016 American Society for Artificial Internal Organs. (E) Wireless optofluidic probe enabling spatiotemporal pharmacological modulation in freely moving animals. Adapted with permission from ref. 273, copyright 2019 Springer Nature. (F) Neural “chemtrode” with staggered herringbone mixer (SHM) for real-time multiplexed neurochemical delivery. Adapted with permission from ref. 275, copyright 2015 Royal Society of Chemistry. (G) Bioresorbable microfluidic cooling implant for reversible nerve conduction block *via* localized thermal modulation. Adapted with permission from ref. 170, copyright 2022 American Association for the Advancement of Science. (H) Wireless ultrasound-readable implant for continuous intra-abdominal pressure monitoring. Adapted with permission from ref. 287, copyright 2020 IEEE. (I) NIR-responsive MXene–hydrogel scaffolds for vascularized skin flap regeneration and VEGF release. Adapted with permission from ref. 298, copyright 2022 Wiley-VCH GmbH.

A milestone in implantable drug delivery was the first human trial of a wireless microchip implant for drug delivery in 2012.^245^ Their microfluidic chip as shown in Fig. 6A, implanted subcutaneously in osteoporotic women, contained sealed reservoirs of parathyroid hormone that could be opened electronically to release precise doses on a programmed schedule. Over 4 months, the chip safely delivered daily doses with pharmacokinetics matching injections, improving bone formation markers without adverse events. Similarly, a drug-loaded micro-reservoir implant (∼6 mm diameter, ∼550 μm depth) was designed to enable magnetically actuated drug delivery, where deformation of an iron-oxide-doped PDMS membrane under a ∼200 mT magnetic field allowed precise and repeatable release.^246^ Several platforms have demonstrated the power of localized, programmable drug delivery. One notable example is the wireless, magnetically actuated microfluidic pump developed for dental implants, which enables localized delivery up to 52 μL of antibiotics or regenerative agents directly at the bone-implant interface to promote osseointegration and prevent infections.^173^ The pump integrates a magnetically (<65 mT) responsive valve system that allows external control of fluid release without the need for batteries or onboard electronics.

In a related advance, a self-powered implantable drug delivery system was demonstrated using a triboelectric nanogenerator (TENG) to harvest biokinetic energy and drive an electrochemical microfluidic pump, achieving flow rates up to 40 μL min^−1^.^247^ In *ex vivo* studies, this platform successfully delivered fluorescent particles into porcine eyes, offering a promising battery-free solution for chronic ocular drug therapies. Similarly, a swarm of autonomous untethered microinjectors was developed to penetrate the gastrointestinal epithelium and deliver insulin systemically in live rats *in vivo*, as shown in Fig. 6B.^248^ Although transient, these devices exemplify how microfluidic architectures in such a tiny origami-based self-folding design can enable minimally invasive drug delivery to internal tissue just using body heat.

Thermally triggered systems offer another promising avenue for rapid therapeutic delivery. Elman *et al.* proposed an implantable microfluidic device that used local heating to boil fluid inside a reservoir, bursting a brittle membrane to deliver drugs in emergency scenarios.^249^ Such systems are particularly valuable for treatments requiring immediate intervention, such as acute cardiovascular or neurological crises. Another promising platform is a low-flow, implantable microfluidic pump that uses thermally actuated gallium phase-change material to deliver infusion rates ranging from 18 nL min^−1^ to 104 nL min^−1^ to sensitive tissues such as the eye, ear, and brain.^250^ The system enables energy-efficient, periodic infusion at nanoliter-per-minute rates, making it well-suited for chronic therapeutic applications.

Microfluidic systems have also been employed in cancer therapy. An implantable drug-screening microdevice was developed to release microdoses of up to 16 different anticancer agents directly into tumor tissue, as shown in Fig. 6C.^251^ Following short-term exposure, the local tissue response to each drug was evaluated, enabling personalized therapy selection without subjecting the patient to systemic toxicity. Further advancements allowed multiplexed testing of immunotherapies in breast cancer models, demonstrating synergistic drug effects identified through localized microfluidic delivery. An electrochemically actuated implantable microfluidic device was developed featuring a single refillable PDMS reservoir and nano-sandwiched Pt/Ti electrodes, enabling programmable drug release at flow rates of 1 to 2.3 μL s^−1^ under applied voltages of 5–9 V.^252^*In vivo* studies in Kunming mice confirmed excellent biocompatibility over 28 days and effective localized delivery of doxorubicin for pancreatic cancer treatment. An important clinical example of targeted drug delivery is the TAR-200 device, a novel implantable system designed for the sustained release of gemcitabine within the bladder lumen for the treatment of bladder cancer.^253^ The device utilizes osmotic pressure to achieve controlled, continuous drug elution over several weeks, maintaining therapeutic drug concentrations at the target site while minimizing systemic exposure. By overcoming the limitations of traditional intravesical therapies, such as rapid drug washout, TAR-200 exemplifies the clinical potential of implantable microfluidic platforms for localized, long-term cancer treatment.

In the field of neuropharmacology, wireless soft optofluidic implants were developed integrating four microfluidic drug reservoirs, each independently controlled by microscale LEDs for precise drug release.^254^ They demonstrated wireless optofluidic neural probes integrating ultrathin (∼80 μm) microfluidic channels and microscale LEDs (100 μm × 100 μm), achieving efficient wireless drug delivery (0.5 μL at ∼5.2 μL min^−1^) and photostimulation in freely behaving animals without significant brain tissue damage. These soft, battery-free devices enabled pharmacological and optical modulation of brain circuits in freely behaving animals, representing a powerful tool for studying behavior and developing therapies for neurological disorders. A lot of devices with combinations of neural interface with drug/chemical agents delivery have been shown by researchers, and we will cover more details in section 4.2.3 as these devices can have dual or more functionality.

The rise of smart, closed-loop microfluidic delivery systems further expands possibilities. Concepts combining biosensors with implantable pumps are emerging, aiming to create autonomous systems that release therapeutics in response to real-time physiological cues, such as glucose levels (for diabetes) or inflammatory markers (for autoimmune diseases).^255–257^ Although most of these remain at the prototype stage, the technological foundation laid by early wireless, magnetically actuated, and thermally triggered microfluidic implants strongly supports future clinical translation.^31^

Many implantable pumps provide incremental improvements in control, volume, or longevity. However, innovations like magnetically actuated refillable reservoirs, triboelectric-powered ocular implants, and thermally triggered burst devices represent transformative strategies. These eliminate batteries, enable remote actuation, and introduce autonomous or emergency-triggered delivery—broadening therapeutic use-cases and reducing surgical burden.

#### 4.2.2. Artificial organs and organ support

Implantable microfluidic devices are playing a pivotal role in the development of artificial organs and organ support systems. Traditional organ transplantation and dialysis technologies, while lifesaving, face significant limitations including organ shortages, immunosuppression requirements, and systemic side effects.^258^ Microfluidic engineering offers innovative solutions by replicating key physiological functions in miniaturized, implantable devices, aiming for long-term autonomous support of organ systems.^259^

One of the most notable advances in this area is the development of bioartificial kidneys using implantable microfluidic filtration systems. An implantable renal assist device was developed incorporating 400 nm-thick polysilicon flat sheet membranes with 5–8 nm, 2 μm slit-shaped silicon nanopore membranes capable of size-selective filtration of waste products while retaining essential proteins and cells for up to 3–8 days.^260^ The device mimics glomerular filtration and, when combined with a bioreactor of renal tubular cells, could replace both the filtration and reabsorption functions of the kidney as shown in Fig. 6D. These devices utilize microfluidic architectures to achieve efficient solute clearance and electrolyte balance while minimizing blood flow resistance. Additionally, work by Humes *et al.* on renal assist devices has laid the groundwork for combining living cells with mechanical filtration.^261^ Innovations such as dialysate regeneration loops, miniaturized closed-circuit designs, and urea removal strategies are now bringing fully implantable kidney replacements closer to clinical reality.^262^ Such systems exemplify how microfluidic architectures can be leveraged to recreate complex organ-level functions within compact, implantable platforms.

Further advancing the field of bioartificial organ systems, Lieberthal *et al.* developed an implantable 3D-printed hydrogel device incorporating a pair of parallel millifluidic channels that function as portal-venous (PV) and hepatobiliary (HB) structures to support liver tissue engineering.^263^ Upon implantation, blood perfusion through the nanoporous hydrogel walls significantly enhanced hepatocyte viability and functional protein secretion, with a flow rate of 5 mL min^−1^ generating approximately 20 dyn cm^−2^ of wall shear stress—within the physiological range observed in human arteries—thereby highlighting the potential of microfluidic architectures to sustain metabolic activity *in vivo* for upto 2 days. This work underscores the feasibility of scalable, implantable liver-mimetic devices for future therapeutic applications. In the field of diabetes management, emerging microfluidic platforms are also advancing pancreatic tissue engineering and endocrine organ support. Similarly, a valve-integrated microfluidic chip was developed to enable dynamic glucose stimulation and insulin secretion collection from a single pancreatic islet, demonstrating precise functional assessment relevant to future implantable bioartificial pancreas systems.^264^

A particularly elegant aspect of these microfluidic organ-support devices is their potential to function without external power sources. Systems relying on passive diffusion, pressure-driven flow, or self-powered chemical reactions minimize the need for battery replacements and reduce surgical risks.^265–267^ Furthermore, the use of bioresorbable or biocompatible materials such as silicon, PEG hydrogels, and PDMS facilitates long-term implantation with minimal immune response.^268–270^ Overall, implantable microfluidic systems for organ support represent a paradigm shift in regenerative medicine. By closely replicating native organ functions within compact, engineered systems, these devices offer transformative potential for treating chronic organ failure, reducing dependency on donor organs, and improving patient survival and autonomy.^95^

While many microfluidic dialysis systems refine membrane performance or reduce footprint, implantable bioartificial organs that integrate living cellular components within microfluidic scaffolds mark a fundamental shift. These systems move beyond passive filtration toward dynamic tissue mimicry and biologically interactive function, setting a transformative precedent in organ replacement.

#### 4.2.3. Neural interfaces and neuromodulation

Implantable microfluidic systems have opened transformative avenues in both neuroscience research and clinical neurotechnology.^271^ Platforms like the lab-on-a-brain developed by Takehara *et al.* exemplify how implantable micro-optical fluidic devices can enable long-term two-photon imaging while locally delivering pharmacological agents into the brain.^166^ By replacing part of the skull with an integrated device, these systems allow repeated chemical interventions at a rate of 10 μL min^−1^ for 20 min and high-resolution imaging of neuronal structures (<1 μm), facilitating studies on synaptic plasticity, memory, and neurodegeneration. This minimally invasive approach preserves the natural microenvironment of the brain and reduces inflammation compared to traditional cannulation techniques.

Beyond imaging, flexible multifunctional probes that combine fluidics with optogenetics and electrophysiology have enabled advanced neural circuit interrogation. Fiber-based neural probes with feature sizes as small as 5 μm have been developed to enable simultaneous optical stimulation, localized drug delivery, and electrophysiological recording. These multifunctional probes are embedded within a soft, stretchable architecture, making them well-suited for chronic implantation and long-term neurophysiological studies.^272^ Similarly, wireless optofluidic systems integrating micro-LEDs and fluidic drug reservoirs, as demonstrated by Jeong *et al.* and Qazi *et al.*, have removed the need for tethered operation, enabling real-time programmable pharmacology and optogenetic stimulation in freely moving animals.^254,273^ Qazi *et al.* demonstrated the wireless optofluidic probe system (Fig. 6E), weighing approximately 2 g and occupying 1260 mm^3^, reliably delivered 0.47 μL of fluid per activation within 12 seconds, while exerting minimal tissue pressure (∼0.77 kPa), ensuring safe chronic pharmacological interventions in freely moving mice.^273^ These platforms allow complex behavioral experiments that require spatiotemporal control over neuronal populations, critical for studying reward, addiction, and mood regulation.

A major advance in implantable microfluidic design is represented by SU-8-based multi-site neural probes that combine high-resolution depth recordings with independent drug delivery channels. This platform enables precise administration of small drug volumes—from nanoliters to microliters—while simultaneously recording local field potentials and single-neuron activity.^274^ Chemical neuromodulation has been further expanded through the development of multiplexed drug delivery platforms. In a similar context, a neural “chemtrode” was developed by integrating a 3-inlet staggered herringbone mixer (SHM) into a silicon microfluidic probe, enabling multiplexed delivery of neuroactive agents at dynamically tunable concentrations through a compact platform (Fig. 6F).^275^ The system achieved rapid switching between drugs with a residence time of approximately 14 seconds and a total swept volume of only ∼66 nL, supporting real-time modulation of neural activity with minimal fluid burden on brain tissue. *In vivo* experiments demonstrated controlled delivery of pilocarpine and tetrodotoxin (TTX) into the hippocampus, allowing reversible modulation of neuronal firing rates during a single implantation. In a related development, flexible penetrating microelectrode arrays (FPMAs) integrated with microfluidic cables were demonstrated to simultaneously record electrophysiological signals and deliver chemical agents.^276^*In vivo* experiments demonstrated effective KCl infusion at a flow rate of 1.4 ± 0.15 μL min^−1^, resulting in an ∼80% increase in neural spiking activity across the electrode array, confirming the platform's utility for modulating brain activity with minimal tissue disruption offering a powerful method to study drug effects on brain networks and to bypass the blood–brain barrier for targeted therapies.

The integration of microfluidics with real-time optical monitoring technologies marks another major application domain. An integrated wireless microfluidic and fiber-photometry platform was introduced to enable simultaneous drug delivery and neural activity recording *via* fluorescence-based indicators.^277^ By coupling fluidic drug delivery with calcium, neurotransmitter, or neuromodulator imaging, these systems enable closed-loop experiments where the immediate biochemical and electrophysiological impacts of pharmacological interventions can be observed *in vivo*. This approach holds enormous potential for dissecting the real-time dynamics of neuromodulatory systems in behavior and disease.

Peripheral nervous system (PNS) applications have also benefited significantly from implantable microfluidics. Hydrogel-based soft agarose-filled microfluidic nerve cuffs have been developed to provide a self-folding, ion-conductive interface that enables safe delivery of direct current nerve blocks (above 75 μA), addressing the electrochemical and mechanical limitations of traditional metallic nerve cuffs.^278^ Furthermore, Reeder *et al.* introduced a soft, bioresorbable microfluidic device capable of reversible peripheral nerve conduction block through localized cooling as shown in Fig. 6G.^170^ The implant wraps around the nerve without sutures and delivers evaporative cooling *via* perfluoro-pentane and dry nitrogen gas. *In vivo* experiments in rats demonstrated a rapid cooling rate of 3 °C s^−1^, with nerve temperatures reaching as low as −1.4 °C and maintained near 3 °C for over 15 minutes. Acute trials showed a 92% reduction in electromyography (EMG) amplitude and a 7-fold increase in mechanical pain threshold, confirming effective analgesia without permanent tissue damage. The device bioresorbed safely within 20–50 days, highlighting its potential as a non-opioid, minimally invasive alternative for post-surgical and neuropathic pain management.

Beyond chemical and electrical modulation, dynamic structural control is also becoming possible. Notably, an inflatable spinal cord stimulator featuring microfluidic channels that allow the implant to expand into a wide paddle-like shape after insertion.^279^ This design enables minimally invasive implantation while achieving broad coverage of the spinal cord, improving therapeutic efficacy for conditions like intractable back pain and muscle spasms. Outside the mammalian system, implantable microfluidics have been used in biohybrid robotics. One such system integrated electrical stimulation and microfluidic neurotransmitter delivery to modulate the flight behavior of *Manduca sexta* moths.^280^ By combining electrical initiation of flight with chemical modulation of wing power, they achieved enhanced flight control and duration, highlighting the broad potential of microfluidic systems not only for biomedical applications but also for cyborg and environmental sensing platforms.

#### 4.2.4. Implantable biosensors and diagnostics

The development of implantable microfluidic biosensors has greatly expanded the capabilities of real-time, continuous physiological monitoring inside the human body. Unlike traditional diagnostics that rely on intermittent blood draws or imaging, implantable biosensors offer dynamic, on-site measurement of critical biomarkers, enabling earlier detection of pathological changes, personalized treatment, and better disease management.^281–285^

One notable area of advancement is pressure sensing within body cavities. Lo *et al.* developed an implantable, refillable ocular drug delivery system that also incorporated pressure regulation features using microfluidics, illustrating the dual potential of therapeutic delivery and diagnostics within a single implant.^286^ More recently, Jiang *et al.* developed an implantable wireless microfluidic pressure sensor for non-invasive monitoring of intra-abdominal pressure (IAP) using ultrasound imaging (Fig. 6H).^287^ The sensor demonstrated a linear sensitivity of 42 kPa mm^−1^ within physiological IAP ranges (0–12.6 kPa), with spatial resolution of 1.2 kPa/30 μm, and maintained functional integrity over 600 actuation cycles without leakage. *Ex vivo* experiments confirmed accurate pressure readings through ∼15 mm of porcine skin, highlighting its potential for wireless, battery-free monitoring in critical care and surgical applications.

Microfluidic biosensors are also being designed for metabolic monitoring and have gained high interest towards autonomic therapy implantable applications.^288^ A microfabricated implantable device was developed combining protected glucose biosensors with electroactive microvalves for controlled insulin release, demonstrating a proof-of-concept for closed-loop, responsive therapeutic systems for conditions such as diabetes and rheumatoid arthritis without the need for frequent blood sampling.^289^

Expanding into orthopedic applications, a conformable microfluidic capacitive force sensor was developed and embedded in trial acetabular cups for hip replacement surgeries to enable real-time force measurement during implantation procedures.^171^ These implants provide surgeons with real-time quantitative feedback on force distribution during joint placement accurately measured forces up to 400 N, significantly improving implant positioning accuracy into curved joint geometries without affecting function and reducing the risk of postoperative complications or early implant failure.

Additionally, researchers are exploring integration of biosensors with therapeutic implants to create smart closed-loop systems. For example, in the field of neuroengineering, an electrocorticography (ECoG) array was demonstrated with integrated microfluidic ion pumps, enabling simultaneous neural recording and localized pharmacological delivery.^290^ Such multifunctional platforms blur the traditional boundaries between diagnostics and therapeutics, representing a major step toward fully autonomous, adaptive implants.

In summary, implantable microfluidic biosensors are redefining medical diagnostics by enabling continuous, real-time physiological monitoring *in situ*. As miniaturization, biocompatibility, and wireless readout technologies advance, these devices are poised to become essential tools not only for early disease detection but also for guiding dynamic, personalized therapeutic interventions.

#### 4.2.5. Tissue engineering and regenerative medicine

Microfluidic platforms have emerged as transformative tools in tissue engineering and regenerative medicine due to their ability to replicate complex biochemical and mechanical microenvironments. These systems enable fine control over nutrient delivery, waste removal, mechano-transduction, and spatial cell patterning—key factors in guiding tissue development and integration.^291–294^ In this section, we highlight representative applications spanning bone, skin, and vascularized tissue regeneration to illustrate how implantable microfluidics can support diverse tissue types and repair strategies.

Mechanical stimulation is a fundamental principle in bone tissue engineering, as bone is a highly mechanosensitive tissue that remodels itself in response to mechanical cues.^295^ Chen *et al.* leveraged this principle by designing an implantable, wireless, magnetically actuated microfluidic pump capable of generating controlled pressure fluctuations within the intramedullary cavity of long bones.^162^ By modulating local fluid flow and inducing cyclic pressure changes, the device mimics the physiological mechanical environment experienced during normal skeletal loading, thereby promoting osteogenic activity without the need for pharmacological agents. This strategy directly targets mechanotransduction pathways to stimulate osteoblast proliferation and matrix mineralization, offering a microfluidic approach to enhancing bone regeneration. Furthermore, the system's wireless actuation and battery-free design minimize invasiveness and improve biocompatibility, making it a promising platform for long-term orthopedic implants aimed at treating bone loss disorders such as osteoporosis.

Implantable microfluidic platforms are revolutionizing tissue engineering and regenerative medicine by offering precise control over the biochemical and mechanical environments essential for tissue development and function. Early strategies focused on flexible, thread-based microfluidic devices capable of embedding sensors and fluidic channels within 3D tissue architectures, allowing real-time monitoring and manipulation of physiological parameters *in vivo*.^174^ These innovations provided a foundation for designing microfluidic scaffolds that intimately integrate with host tissues, enabling localized sensing, therapeutic delivery, and tissue remodeling.

Biomaterial innovation has been a critical driver in advancing implantable microfluidic scaffolds. Polymers such as polydimethylsiloxane (PDMS), poly(lactic-*co*-glycolic acid) (PLGA), and natural hydrogels have been widely adopted to create biocompatible and functionalized scaffolds that mimic the extracellular matrix.^296,297^ The integration of microfluidics with polymeric materials allows the fabrication of dynamic, biomimetic structures capable of guiding cellular growth, nutrient delivery, and waste removal. Notably, dynamically responsive hydrogel scaffolds were developed for skin flap regeneration, composed of MXene-incorporated poly(NIPAM) hydrogels that exhibited near-infrared (NIR)-responsive shrinkage. As shown in Fig. 6I, the scaffolds achieved up to 55% volume reduction under 46 °C heating, facilitating cell infiltration and controlled VEGF release.^298^*In vivo* mouse studies demonstrated that VEGF-loaded scaffolds under NIR irradiation significantly improved skin flap survival (reducing necrosis rates to 17.9% compared to 63.7% in controls), enhanced angiogenesis, decreased inflammation, and attenuated apoptosis, highlighting the therapeutic potential of microfluidically printed, stimulus-responsive implants.

A significant breakthrough in this field has been the development of scaffolds with perfusable microvascular networks. Devices like AngioChip and biodegradable microvessel frameworks have shown that integrating endothelialized microchannels within biodegradable matrices can support large, metabolically active tissues by maintaining nutrient perfusion and promoting vascular integration upon implantation.^299,300^ Similarly, Kim *et al.* demonstrated that implantable PLGA microfluidic devices seeded with human endothelial progenitor cells could facilitate the *in vivo* formation of functional capillary networks, crucial for engineered tissue survival.^301^

Overall, implantable microfluidic scaffolds represent a critical advancement in regenerative medicine, enabling the construction of vascularized, functional tissues across a range of organ systems. These platforms not only support cell viability and integration but also offer dynamic responsiveness, controlled therapeutic delivery, and scalable fabrication methods, paving the way for future clinical applications in organ replacement and regenerative therapies.^302^

## 5. Conclusion and future perspectives

Microfluidic technologies have catalyzed transformative advances in the development of wearable and implantable biomedical devices, enabling non-invasive monitoring, localized therapy, and personalized health management.^31,106^ The integration of soft materials, miniaturized sensors, and scalable fabrication strategies has facilitated the realization of skin-conformal patches and implantable microsystems capable of direct interfacing with biological fluids.^10,303^ Applications now span a broad range, including sweat-based fitness tracking, implantable drug delivery systems, biosensors for neurological monitoring, and microfluidic scaffolds for tissue engineering and regenerative medicine.

Despite this rapid progress, several critical challenges remain before widespread clinical translation can be achieved. For wearable systems, maintaining long-term reliability under dynamic physical and chemical conditions is a persistent obstacle. Variability in biofluid composition, pH fluctuations, and biofouling can impair sensor accuracy, while mechanical deformation and adhesion fatigue can compromise device durability.^9^ Electrochemical sensors, particularly those based on irreversible binding reactions, often suffer from regeneration limitations and signal drift.^176^ The integration of soft–rigid interfaces, such as between flexible microfluidics and embedded electronics, remains mechanically vulnerable. Furthermore, most wearable sensors are limited to detecting low-molecular-weight analytes; the reliable detection of macromolecules (*e.g.*, cytokines or proteins) remains difficult due to their low abundance and limited correlation with systemic biomarkers in peripheral biofluids.^304,305^

For implantable systems, key barriers include long-term biocompatibility, immune responses, material degradation, and power autonomy.^306^ Many current devices depend on external power supplies or rigid components, hindering miniaturization and patient comfort. Importantly, clinical translation faces regulatory constraints, including compliance with FDA regulations and ISO standards.^307^ Most such devices are classified as Class III by the FDA, requiring rigorous Premarket Approval (PMA) that includes extensive evidence of biocompatibility (ISO 10993), manufacturing quality (ISO 13485), and electrical safety (IEC 60601-1). In addition to technical validation, manufacturers must also demonstrate long-term safety, sterility assurance, and reproducibility across patient populations. Furthermore, lack of established reimbursement codes and limited clinical familiarity with microfluidic systems can slow adoption, underscoring the need for collaborative engagement with regulatory agencies, clinicians, and health economists.

Bridging this gap will require focused efforts along several converging fronts. Advances in material science are paramount, particularly the development of antifouling, self-healing, and bioresorbable substrates that ensure long-term stability while minimizing chronic immune response. Emerging biodegradable platforms capable of full resorption within the body offer a promising pathway for temporary implants without retrieval procedures. To support autonomous operation, energy harvesting technologies—such as enzymatic biofuel cells, triboelectric nanogenerators, and hybrid power systems—should be integrated to replace or supplement traditional battery modules. Smarter fluidic architectures incorporating elastofluidic logic, capillary burst valves, and droplet manipulation can enable more precise and programmable fluid handling under physiological conditions.^308^

A particularly promising direction lies in the integration of artificial intelligence and machine learning algorithms with microfluidic sensors to enable real-time, on-device data interpretation and predictive diagnostics.^309–311^ Coupling such analytics with closed-loop control systems could usher in a new era of personalized therapy, allowing wearable or implantable devices to autonomously respond to changes in a patient's physiological state. Furthermore, scalable and modular manufacturing approaches—such as roll-to-roll printing, soft lithography, and 3D hybrid integration—will be essential for transitioning from benchtop prototypes to mass-producible and clinically deployable products.

Looking ahead, wearable and implantable microfluidic platforms are poised to play a central role in decentralized and precision healthcare. However, their success will depend not only on continued innovation in materials, electronics, and system integration, but also on early-stage alignment with regulatory requirements and clinical needs. Multidisciplinary collaboration among engineers, clinicians, regulators, and industry stakeholders will be vital to navigate the complex pathway from lab discovery to bedside adoption. With thoughtful design and translational foresight, these technologies have the potential to revolutionize disease monitoring, treatment personalization, and long-term health management.

## Conflicts of interest

There are no conflicts to declare.

## Data Availability

No new data was generated or analyzed in this study. All data discussed are from previously published sources cited in the manuscript.
